# Amplification of the PLAG-family genes—*PLAGL1* and *PLAGL2*—is a key feature of the novel tumor type *CNS embryonal tumor with PLAGL amplification*

**DOI:** 10.1007/s00401-022-02516-2

**Published:** 2022-11-27

**Authors:** Michaela-Kristina Keck, Martin Sill, Andrea Wittmann, Piyush Joshi, Damian Stichel, Pengbo Beck, Konstantin Okonechnikow, Philipp Sievers, Annika K. Wefers, Federico Roncaroli, Shivaram Avula, Martin G. McCabe, James T. Hayden, Pieter Wesseling, Ingrid Øra, Monica Nistér, Mariëtte E. G. Kranendonk, Bastiaan B. J. Tops, Michal Zapotocky, Josef Zamecnik, Alexandre Vasiljevic, Tanguy Fenouil, David Meyronet, Katja von Hoff, Ulrich Schüller, Hugues Loiseau, Dominique Figarella-Branger, Christof M. Kramm, Dominik Sturm, David Scheie, Tuomas Rauramaa, Jouni Pesola, Johannes Gojo, Christine Haberler, Sebastian Brandner, Tom Jacques, Alexandra Sexton Oates, Richard Saffery, Ewa Koscielniak, Suzanne J. Baker, Stephen Yip, Matija Snuderl, Nasir Ud Din, David Samuel, Kathrin Schramm, Mirjam Blattner-Johnson, Florian Selt, Jonas Ecker, Till Milde, Andreas von Deimling, Andrey Korshunov, Arie Perry, Stefan M. Pfister, Felix Sahm, David A. Solomon, David T. W. Jones

**Affiliations:** 1 Hopp Children’s Cancer Center Heidelberg (KiTZ), Im Neuenheimer Feld 280, 69120 Heidelberg, Germany; 2 Division of Pediatric Glioma Research (B360), German Cancer Research Center (DKFZ), Im Neuenheimer Feld 280, 69120 Heidelberg, Germany; 3 Division of Pediatric Neurooncology, German Cancer Consortium (DKTK), German Cancer Research Center (DKFZ), Heidelberg, Germany; 4 Department of Neuropathology, Institute of Pathology, University Hospital Heidelberg, Heidelberg, Germany; 5 Clinical Cooperation Unit Neuropathology, German Consortium for Translational Cancer Research (DKTK), German Cancer Research Center (DKFZ), Heidelberg, Germany; 6 Faculty of Biosciences, Heidelberg University, Heidelberg, Germany; 7 Institute of Neuropathology, University Medical Center Hamburg-Eppendorf, Hamburg, Germany; 8 Geoffrey Jefferson Brain Research Centre, Division of Neuroscience and Experimental Psychology, Faculty of Biology, Medicine and Health, University of Manchester, Manchester, UK; 9 Department of Radiology, Alder Hey Children’s NHS Foundation Trust, Liverpool, UK; 10 Division of Cancer Sciences, University of Manchester, Manchester Academic Health Science Centre, Manchester, UK; 11 Department of Pediatric Hematology and Oncology, Alder Hey Children’s NHS Foundation Trust, Liverpool, UK; 12 Princess Máxima Center for Pediatric Oncology, Utrecht, The Netherlands; 13 Department of Pathology, Amsterdam University Medical Centers, Location VUmc and Brain Tumor Center Amsterdam, Amsterdam, The Netherlands; 14 Department of Pediatric Oncology and Hematology, Skåne University Hospital, Lund University, Lund, Sweden; 15 Department of Oncology-Pathology, Karolinska Institutet, Stockholm, Sweden; 16 Prague Brain Tumor Research Group, Second Faculty of Medicine, Charles University and University Hospital Motol, Prague, Czech Republic; 17 Department of Pediatric Haematology and Oncology, Second Faculty of Medicine, Charles University and University Hospital Motol, Prague, Czech Republic; 18 Department of Pathology and Molecular Medicine, Second Faculty of Medicine, Charles University and University Hospital Motol, Prague, Czech Republic; 19 Institut de Pathologie Multisite-Site Est, Groupement Hospitalier Est, Hospices Civils de Lyon, Lyon, France; 20 Department of Pediatric Oncology and Hematology, Charité-Universitätsmedizin Berlin, Corporate Member of Freie Universität Berlin, Humboldt-Universität zu Berlin, and Berlin Institute of Health, Berlin, Germany; 21 Department of Pediatric Hematology and Oncology, University Medical Center Hamburg-Eppendorf, Hamburg, Germany; 22 Research Institute Children’s Cancer Center Hamburg, Hamburg, Germany; 23 University of Bordeaux, Bordeaux Institute of Oncology (BRIC)-INSERM U1312 Université de Bordeaux, 146 rue Leo Saignat, Case 76, 33076 Bordeaux, France; 24 Aix-Marseille Univ, APHM, CNRS, INP, Inst Neurophysiopathol, CHU Timone, Service d’Anatomie Pathologique et de Neuropathologie, Marseille, France; 25 Division of Pediatric Hematology and Oncology, University Medical Center Göttingen, Göttingen, Germany; 26 Department of Pediatric Oncology, Hematology, Immunology and Pulmonology, University Hospital Heidelberg, Heidelberg, Germany; 27 Department of Pathology, Rigshospitalet, Copenhagen, Denmark; 28 Department of Clinical Pathology, Kuopio University Hospital and Unit of Pathology, Institute of Clinical Medicine, University of Eastern Finland, Kuopio, Finland; 29 Department of Pediatrics, Pediatric Hematology and Oncology Ward, Kuopio University Hospital and Institute of Clinical Medicine, University of Eastern Finland, Kuopio, Finland; 30 Department of Pediatrics and Adolescent Medicine, Comprehensive Cancer Center and Comprehensive Center for Pediatrics, Medical University of Vienna, 1090 Vienna, Austria; 31 Division of Neuropathology and Neurochemistry, Department of Neurology, Medical University of Vienna, Vienna, Austria; 32 Division of Neuropathology, National Hospital for Neurology and Neurosurgery, University College London Hospitals NHS Foundation Trust, Queen Square, London, UK; 33 Department of Neurodegenerative Disease, UCL Queen Square Institute of Neurology, Queen Square, London, UK; 34 Department of Developmental Biology and Cancer, UCL GOS Institute of Child Health, University College London, London, UK; 35 Murdoch Children’s Research Institute and Department of Paediatrics, University of Melbourne, Royal Children’s Hospital, Melbourne, Australia; 36 Department of Pediatric Oncology/Hematology/Immunology, Olgahospital, Klinikum Stuttgart, Stuttgart, Germany; 37 Department of Developmental Neurobiology, St. Jude Children’s Research Hospital, Memphis, TN USA; 38 Department of Pathology and Laboratory Medicine, The University of British Colombia, Vancouver, Canada; 39 Department of Pathology, NYU Langone Medical Center, New York, NY USA; 40 Department of Pathology and Laboratory Medicine, The Aga Khan University, Karachi, Pakistan; 41 Department of Pediatric Hematology-Oncology, Valley Children’s Hospital, Madera, CA USA; 42 Clinical Cooperation Unit Pediatric Oncology, German Consortium for Translational Cancer Research (DKTK), German Cancer Research Center (DKFZ), Heidelberg, Germany; 43 KiTZ Clinical Trial Unit (ZIPO), Department of Pediatric Hematology and Oncology, Heidelberg University Hospital, Heidelberg, Germany; 44 Division of Neuropathology, Department of Pathology, University of California San Francisco (UCSF), 513 Parnassus Ave, Health Sciences West 451, San Francisco, CA 94143 USA

**Keywords:** PLAGL1, PLAGL2, Molecular neuro-oncology, Pediatric cancer

## Abstract

Pediatric central nervous system (CNS) tumors represent the most common cause of cancer-related death in children aged 0–14 years. They differ from their adult counterparts, showing extensive clinical and molecular heterogeneity as well as a challenging histopathological spectrum that often impairs accurate diagnosis. Here, we use DNA methylation-based CNS tumor classification in combination with copy number, RNA-seq, and ChIP-seq analysis to characterize a newly identified CNS tumor type. In addition, we report histology, patient characteristics, and survival data in this tumor type. We describe a biologically distinct pediatric CNS tumor type (*n* = 31 cases) that is characterized by focal high-level amplification and resultant overexpression of either *PLAGL1* or *PLAGL2*, and an absence of recurrent genetic alterations characteristic of other pediatric CNS tumor types. Both genes act as transcription factors for a regulatory subset of imprinted genes (IGs), components of the Wnt/β-Catenin pathway, and the potential drug targets *RET* and *CYP2W1*, which are also specifically overexpressed in this tumor type. A derived PLAGL-specific gene expression signature indicates dysregulation of imprinting control and differentiation/development. These tumors occurred throughout the neuroaxis including the cerebral hemispheres, cerebellum, and brainstem, and were predominantly composed of primitive embryonal-like cells lacking robust expression of markers of glial or neuronal differentiation (e.g., GFAP, OLIG2, and synaptophysin). Tumors with *PLAGL1* amplification were typically diagnosed during adolescence (median age 10.5 years), whereas those with *PLAGL2* amplification were diagnosed during early childhood (median age 2 years). The 10-year overall survival was 66% for *PLAGL1*-amplified tumors, 25% for *PLAGL2*-amplified tumors, 18% for male patients, and 82% for female patients. In summary, we describe a new type of biologically distinct CNS tumor characterized by *PLAGL1/2* amplification that occurs predominantly in infants and toddlers (*PLAGL2*) or adolescents (*PLAGL1*) which we consider best classified as a CNS embryonal tumor and which is associated with intermediate survival. The cell of origin and optimal treatment strategies remain to be defined.

**Supplementary Information:**

The online version contains supplementary material available at 10.1007/s00401-022-02516-2.

## Introduction

The current 2021 edition of the World Health Organization (WHO) Classification of Central Nervous System (CNS) Tumors comprises more than 100 distinct pediatric and adult tumor types based on combined phenotypic and genotypic classification [1]. This updated classification is reflective of the notion that adult-type and pediatric-type tumors are markedly different and can be distinguished based on their molecular features, as is the case for diffuse gliomas, such as the H3 G34-mutant diffuse hemispheric glioma or the H3 K27-altered diffuse midline glioma. The latter is predominant in pediatric patients and restricted to certain anatomic locations [25, 26, 48, 68]. While gliomas constitute the majority of malignant CNS tumors, further CNS tumor categories such as glioneuronal and neuronal tumors, embryonal tumors, pineal tumors, and mesenchymal tumors also contribute substantially to mortality [35, 40]. According to the latest report from the CBTRUS, the CNS is the most common cancer site in children aged 0–14 years, and CNS tumors are the most common cause of cancer-related death in this age group [40]. Considerable heterogeneity can be found between the different tumor types in terms of both molecular alterations and clinical outcomes. Molecular analyses classify ependymomas, for example, into at least ten subgroups despite shared histological features, and revealed that medulloblastomas comprise multiple distinct molecular groups and subgroups [11, 33, 52]. In addition to the above-mentioned H3-altered gliomas, extensive heterogeneity in IDH- and histone H3-wild-type pediatric high-grade gliomas (HGG) has been observed, with multiple subgroups displaying differential enrichment of affected oncogenes and clinical features [14, 60]. Such molecular heterogeneity, together with a broad histological spectrum of many CNS tumors, can make histopathological diagnosis highly challenging and observer-dependent. To address this, a DNA methylation-based CNS tumor classification system was developed, seeking to improve diagnostic accuracy and objectivity across all CNS tumor types and age groups [12]—a principle which was widely adopted by the latest WHO classifications [35, 41].

Here, we utilized a broad genome-wide DNA methylation cohort, combined with copy number profiling, targeted next-generation DNA sequencing, and RNA sequencing, to identify a rare CNS tumor type characterized by amplification and overexpression of either *PLAGL1* (located at chromosome 6q24.2) or *PLAGL2* (located at chromosome 20q11.21). The pleomorphic adenoma gene (PLAG) family of transcription factors (TFs) comprises three genes, namely *PLAG1*, *PLAGL1*, and *PLAGL2*, whose roles are multifaceted and dependent on their different DNA-binding capacities as well as on the cellular context [20]. While *PLAGL1* has been suggested as a putative tumor suppressor gene, *PLAG1* and *PLAGL2* are presumed proto-oncogenes [4]. In the brain tumor context, our evidence supports an oncogenic role also for *PLAGL1*, which is further substantiated by the recent discovery of recurrent *PLAGL1* fusions in a subset of pediatric-type supratentorial neuroepithelial tumors [54].

Using ChIP-seq data, we show that both PLAGL1 and PLAGL2 act as TFs for the kinase *RET* that is specifically overexpressed in our cohort of *PLAGL1/2*-amplified tumors. RET functions as an oncogenic driver in a variety of cancers and serves as a therapeutic target, with selective RET inhibitors showing promising results in patients [51, 57–59]. We also demonstrate that PLAGL1 and PLAGL2 potentially act as TFs for components of the Wnt/β-Catenin pathway and a set of imprinted genes (IG) that was shown to be controlled by Plagl1 and to regulate the imprinted gene network (IGN) in mouse models [36, 63, 64]; this set of genes, including *IGF2*, is also deregulated in the PLAGL-amplified tumors. In addition, we derive a gene-signature (*n* = 250) characteristic for PLAGL-amplified tumors that indicates dysregulation of imprinting control and differentiation/development as a prominent feature, and shed light on the molecular, histopathologic, and clinical parameters of this novel CNS tumor type.

## Materials and methods

### Patients and samples

Patient samples and retrospective clinical information were either obtained through the databases of the Heidelberg University Department of Neuropathology and the German Cancer Research Center (DKFZ), or directly collected from the respective collaborating national or international institutions in compliance with local regulations. The sample set included CNS tumors enrolled in the INFORM, PTT2.0, and MNP2.0 molecular profiling studies [50, 67].

### Histology and immunohistochemistry

Detailed histopathological review was retrospectively performed on a subset of the tumors (*n* = 15) to investigate their histological and immunohistochemical features. Representative hematoxylin and eosin (H&E)-stained sections and immunohistochemical stains from the 15 tumors were digitally scanned on an Aperio slide scanner to assemble a digital pathology library from which histological and immunohistochemical features were reviewed and annotated using ImageScope software (Leica Biosystems). H&E and immunohistochemical staining was performed at the UCSF Histology Laboratory and the Heidelberg University Department of Neuropathology or received from other participating medical centers. Immunohistochemistry was performed on formalin-fixed, paraffin-embedded tissue sections using the following antibodies: glial fibrillary acidic protein (GFAP, DAKO, polyclonal, 1:3000 dilution, no antigen retrieval), oligodendrocyte transcription factor 2 (OLIG2, Immuno Bio Labs, polyclonal, 1:200 dilution, ER1 antigen retrieval), synaptophysin (Cell Marque, polyclonal, 1:100 dilution, ER2 antigen retrieval), neurofilament (Cell Marque, clone 2F11, undiluted, ER1 antigen retrieval), epithelial membrane antigen (EMA, Leica, clone GP1.4, undiluted, ER1 antigen retrieval), S100 (DAKO, polyclonal, 1:2,000 dilution, no antigen retrieval), CD99 (Signet, clone CD99, 1:400 dilution, ER1 antigen retrieval), BCOR (Santa Cruz Biotechnology, sc-514576, clone C-10, 1:200 dilution, ER2 antigen retrieval), desmin (Cell Marque, clone D33, undiluted, ER1 antigen retrieval or DAKO, M0760, clone D33, 1:50 dilution, CC1 antigen retrieval), GAB1 (Abcam, ab27439, polyclonal, 1:50 dilution, CC2 antigen retrieval), YAP1 (Cell Signaling, #14074, clone D8H1X, 1:100 dilution), beta-catenin (BD Bioscience, 610153, clone 14, 1:100 dilution, CC1 antigen retrieval), and Ki-67 (Dako, clone Mib1, 1:50 dilution, ER2 antigen retrieval). Immunostaining was performed in Leica BOND-III or Ventana BenchMark Ultra automated stainers. Diaminobenzidine was used as the chromogen, followed by hematoxylin counterstain. Scanned image files of H&E and select immunostained sections from a subset of the tumors in this cohort are available for downloading and viewing at the following link: https://figshare.com/projects/CNS_embryonal_tumor_with_PLAGL_amplification/151806.

### Extraction of DNA/RNA

DNA and RNA of samples processed in Heidelberg were extracted using the automated Maxwell nucleic acid purification platform (Promega, Madison, WI, USA). RNA was extracted from fresh–frozen tissue samples with the Maxwell RSC simply RNA Tissue kit and DNA was extracted from fresh–frozen or FFPE tissue samples with the Maxwell RSC Tissue DNA kit or the Maxwell RSC DNA FFPE kit, respectively, according to the manufacturer’s instructions. Other external samples were extracted according to standard local procedures with corresponding QC measures.

### Genome-wide DNA methylation profiling

Fresh–frozen or formalin-fixed paraffin-embedded (FFPE) tissue samples were subjected to genome-wide DNA methylation profiling and were either processed at the DKFZ Genomics and Proteomics Core Facility using the Infinium Methylation EPIC (EPIC) BeadChip or Infinium Human Methylation 450 k Bead Chip arrays (Illumina) according to the manufacturer’s instructions, or at the University of California, San Francisco (UCSF) using the same arrays and methodology. A subset of cases were obtained through uploads to the online http://www.molecularneuropathology.org platform. Methylation array processing was performed as previously described [28]. t-Distributed Stochastic Neighbor Embedding (t-SNE) dimensionality reduction as well as copy number variation (CNV) analysis based on the raw intensities of the methylation array probes were performed as described before [12]. The raw methylation array data of the ET, PLAGL samples have been deposited in NCBI’s Gene Expression Omnibus [16] and are accessible through GEO Series accession number GSE212621 (https://www.ncbi.nlm.nih.gov/geo/query/acc.cgi?acc=GSE212621).

### Copy number analysis

The Integrative Genomics Viewer (IGV) was used to visualize copy number variants (CNVs) of the respective amplified *PLAG*-family genes [46]. Amplifications and deletions were visualized across the entire genome by summary plots. Copy number profiles generated by the conumee R-package were segmented with the circular binary segmentation (CBS) algorithm using the default settings of the conumee package for the 450 k and EPIC array [23]. Resulting segments for each sample were combined to a cohort file using R version 3.6.2 [42], which was then analyzed with the GISTIC_2.0 method available within the GenePattern cloud tool (https://cloud.genepattern.org/) [37, 43]. Human_Hg19.mat was used as reference gene file. Maxspace was set to 10,000. Amplification/deletion thresholds were set to 0.1 and focal length cutoff to distinguish broad from focal events was set to 0.5 (fraction of chromosome arm). Gene GISTIC algorithm was used to calculate the significant regions of deletion. A confidence level of 0.99 as well as a false discovery rate (FDR) *q* value of < 0.25 were used for a region to be considered as significant. Join segment size was set to 4, cap values were set to 1.5, and the maximum number of segments allowed per sample was 2000. Arm level peel off was performed to reduce noise. Amplifications/deletions were rated (0, 1, 2) and divided into three different classes: no amplification/deletion (0; log_2_ ratio < 0.1/log_2_ ratio > − 0.1), low-level amplification (1; 0.1 < log_2_ ratio < 0.9) or low-level deletion (1; − 0.1 > log_2_ ratio > − 1.3), high-level amplification (2; log_2_ ratio > 0.9) or high-level deletion (2; log_2_ ratio < − 1.3). All focal regions and genes identified by GISTIC2.0 are summarized in Supplementary Tables S2, S3, and S4.

### Targeted next-generation DNA sequencing

Genomic DNA extracted from formalin-fixed, paraffin-embedded tumor tissue or frozen tissue was used for targeted next-generation DNA sequencing (NGS) at the UCSF, DKFZ (NPHD gene panel), and PMC for a subset of the patients. For 6 patients (A108, A110, A112, A113, A387, A388), capture-based NGS was performed using the UCSF500 NGS Panel that targets all coding exons of 479 cancer-related genes, select introns and upstream regulatory regions of 47 genes to enable detection of structural variants including gene fusions, and DNA segments at regular intervals along each chromosome to enable genome-wide copy number and zygosity analysis, with a total sequencing footprint of 2.8 Mb [27, 38]. For 5 patients (A93, A94, A96, A379, A380), targeted NGS was performed using the NPHD gene panel developed at the Neuropathology department of the University Hospital Heidelberg that targets the coding exons of 201 cancer-related genes, 9 gene fusions, and 1 upstream regulatory region. For three additional patients (A100, A105, A390), complete exome sequencing was performed. NGS data may be made available upon request.

### RNA sequencing and analysis

All RNAs used for quantitative gene expression analysis were extracted from fresh–frozen tissues. Quality of input RNA was assessed using the Agilent Bioanalyzer System and transcriptome analysis was performed using Illumina TruSeq strand-specific PolyA + libraries on an Illumina HiSeq4000 or NovaSeq device. Differential gene expression analysis was performed with the R2 Genomics Analysis and Visualization Platform (http://r2.amc.nl) using a reference cohort of embryonal tumors (*n* = 117), glial tumors (*n* = 126), and normal fetal and adult brain tissues (*n* = 36) that had been processed the same way. TPM values of the ET, PLAGL samples from our cohort are provided in the supplementary materials (Supplementary Table S8).

### ChIP-seq analysis

Chromatin immunoprecipitation (ChIP) was performed at Active Motif (Carlsbad, CA, USA) using antibodies against H3K27ac (AM#39133, Active Motif), PLAGL1 (HPA055706, Sigma), and PLAGL2 (SAB3500815, Sigma) according to Active Motif’s established ChIP protocol, which includes validation of ChIP reactions via quantitative PCR (qPCR). Twenty pooled CNS tumor samples were used as an Input control. Illumina sequencing libraries were prepared from ChIPs and Input at Active Motif using their standard protocol. Libraries were sequenced via paired-end sequencing with a read length of 100 bp or 75 bp at the DKFZ Genomics and Proteomics Core Facility on the Illumina HiSeq 4000 or NextSeq550, respectively.

Paired end reads were aligned to hg38 using bowtie2. Alignment SAM files were converted to BAM files using samtools. Sambamba was used to sort and remove multimapped, unmapped and duplicated reads from the resulting BAM files. MACS2 was used to call narrow peaks on sorted and processed BAMS with input as the reference. Processed BAM files were normalized with bamCompare (—normalizeUsing BPM—scaleFactorsMethod None—smoothLength 60—extendReads—centerReads) using input as reference for visual comparison of ChIP-seq signal around gene loci.

### Survival analysis

Survival analysis was performed using R version 3.6.2 [42]. The Kaplan–Meier method was used to determine overall survival (OS) and progression-free survival (PFS) for the *PLAGL1-* and *PLAGL2*-amplified tumors separately, as well as stratified by sex. The log-rank test (p-value) was used to identify differences between the Kaplan–Meier curves. Overall survival was defined as the time between first diagnosis and last follow-up date or death, and PFS was defined as the time between first diagnosis and time point of first relapse. A swimmer plot was used to display survival times, treatment, and outcome for each patient.

## Results

### Methylation analysis

**Fig. 1:**
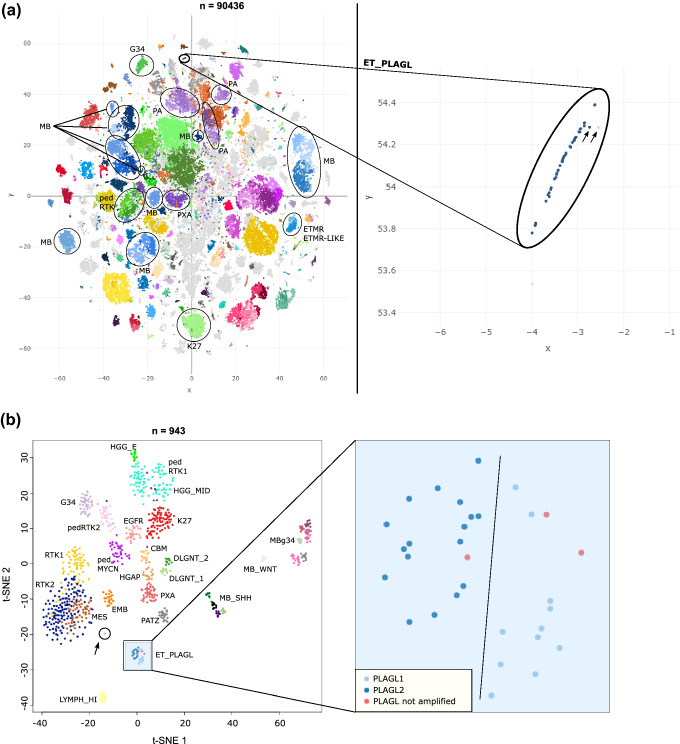
DNA methylation clustering identifies a novel epigenetically distinct subtype of CNS embryonal tumor characterized by focal PLAG-family gene amplification. **a** Left: DNA methylation-based t-SNE analysis of > 90,000 pediatric and adult tumor samples. Circled are different medulloblastoma (MB) and embryonal tumor with multilayered rosettes (ETMR) subtypes, the ET, PLAGL type, and various low grade and high grade glioma subtypes—pilocytic astrocytoma (PA), pleomorphic Xanthoastrocytoma (PXA), H3 G34-mutant diffuse hemispheric glioma (G34), H3 K27-altered diffuse midline glioma (K27), diffuse pediatric-type high grade glioma, RTK subtype (pedRTK). Right: enlarged depiction of samples belonging to the ET, PLAGL type. The arrows mark two slightly outlying samples based on t-SNE. Methylation classes are color-coded as described in [12], grey color means the sample could not be matched to any of the existing methylation classes. **b** DNA methylation-based analysis using t-SNE dimensionality reduction on 33 ET, PLAGL tumors and a reference cohort of 910 different CNS tumors including 780 gliomas/glioneuronal tumors and 130 medulloblastomas. Methylation classes are color-coded and labeled using the respective group abbreviations. ET, PLAGL tumors are differentially colored according to their amplified PLAG-family gene. Two outlying ET, PLAGL samples are circled and marked with an arrow. Samples belonging to the ET, PLAGL type are depicted enlarged on the right. Full group names are: adult-type diffuse high grade glioma, IDH-wild type, subtype E (HGG_E), diffuse pediatric-type high grade glioma, RTK1 and 2 subtype (pedRTK1, pedRTK2), HGG-IDH wild type-subclass midline (HGG_MID), diffuse hemispheric glioma, H3 G34-mutant (G34), diffuse midline glioma, H3 K27-altered, subtype EGFR-altered (EGFR), diffuse midline glioma, H3 K27-altered (K27), glioblastoma, IDH-wild type, subtype posterior fossa (CBM), Glioblastoma, IDH-wild type, RTK1 and 2 subtype (RTK1, RTK2), Glioblastoma, IDH-wild type, mesenchymal subtype (MES), diffuse pediatric-type high grade glioma, MYCN subtype (pedMYCN), embryonal tumor, not otherwise specified (EMB), high-grade astrocytoma with piloid features (HGAP), Pleomorphic Xanthoastrocytoma (PXA), diffuse leptomeningeal glioneuronal tumor, subtype 1 and 2 (DLGNT_1, DLGNT_2), Medulloblastoma, SHH-activated (MB_SHH), Medulloblastoma, WNT-activated (MB_WNT), Medulloblastoma, non-WNT/non-SHH, Group 3 and 4 subtype (MBg34), Inflammatory microenvironment (LYMPH_HI), neuroepithelial tumor with PATZ1 fusion (PATZ), embryonal tumor with PLAG-family gene amplification (ET, PLAGL)

Unsupervised visualization of genome-wide DNA methylation data using t-distributed stochastic neighbor embedding (t-SNE) of > 90,000 pediatric and adult tumor samples of numerous types revealed a subset of 46 tumor samples clustering closely together, but away from established DNA methylation reference classes. Investigation of copy number alterations in each sample indicated amplification of the genetic loci corresponding to one of two different PLAG-family genes (*PLAGL1* at 6q24.2 or *PLAGL2* at 20q11.21) in the majority of tumors—a genetic aberration not known to be a characteristic feature in any of the currently defined CNS tumor types. We, therefore, provisionally named this novel DNA methylation class “CNS embryonal tumor with PLAG-family gene amplification”—ET, PLAGL (Fig. 1a).

Out of the 46 samples initially belonging to the ET, PLAGL cluster, 11 samples were found to be duplicate or relapse samples based on genotype matches. One additional sample was excluded based on quality control indicating array hybridization issues. Another tumor with primary extracranial location was also excluded. This resulted in a set of 33 individual tumors classified as ET, PLAGL that were subjected to further analysis. Including information about the *PLAGL1/PLAGL2* amplification status of each sample, we repeated t-SNE analysis using a select subset of 910 reference tumors of various types—including HGGs, medulloblastomas, and a set of the recently published neuroepithelial tumors with *PATZ1* fusions [6]—together with the 33 ET, PLAGL tumors (Fig. 1b). All ET, PLAGL tumors formed one distinct cluster regardless of their PLAG gene amplification status, which confirmed their group affiliation and epigenetic similarity. The ET, PLAGL cluster was not located in proximity to any of the HGG, medulloblastoma, or other embryonal tumor clusters (Fig. 1a, b) underlining its epigenetic divergence from those tumors—an important point to stress since apart from HGG, medulloblastoma or other embryonal tumors were frequently among the initial histopathological diagnoses for the PLAGL-amplified cases, especially when occurring in the cerebellum. Two samples were found to be outliers that clustered close to ET, PLAGL, but slightly apart from the core group (Fig. 1a) as well as further apart in the refined t-SNE analysis (Fig. 1b). Both outlying samples were *PLAGL1*-amplified tumors, one of which was from an adult patient (age 59 years) and one with unknown age. These two samples were subsequently excluded and the remaining analyses were focused on the core cluster of 31 samples (Fig. 1b). When investigating possible further substructure within this cluster, there was some evidence that the ET, PLAGL cluster could potentially be subdivided into two different sub-clusters based on their location on the t-SNE plot, separating the *PLAGL1*-amplified from the *PLAGL2*-amplified samples. Three samples without apparent PLAG-family gene amplification were also part of the core group based on their DNA methylation pattern, with two seemingly *PLAGL1*-like and one *PLAGL2*-like. In a further t-SNE analysis, which also included a set of the recently published supratentorial ependymoma-like tumors with *PLAGL1* fusions [54], as well as 1,124 sarcomas in addition to the previous reference cohort of 910 tumors, the PLAGL-amplified samples maintained its own unique cluster (Supplementary Fig. 1).

### Copy number analysis

**Fig. 2:**
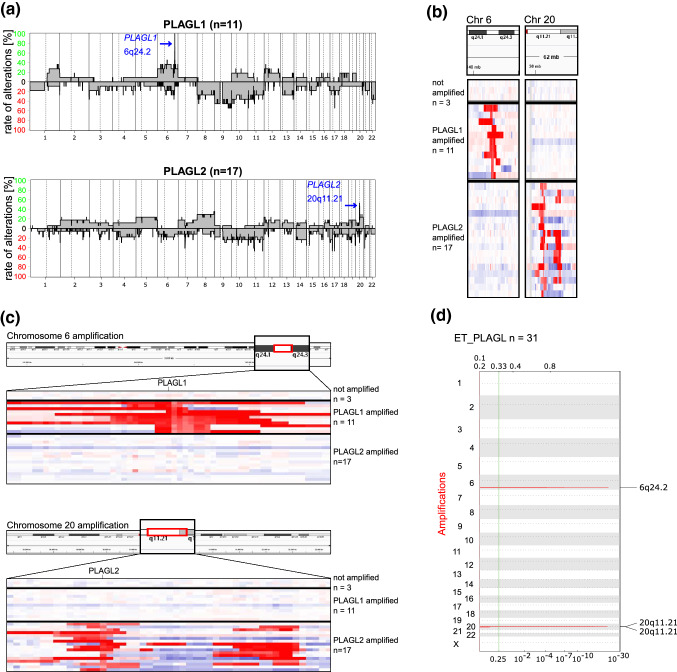
Copy number analysis of CNS embryonal tumors with PLAGL gene amplification. **a** Copy number summary plots were derived per subgroup for *PLAGL1*-amplified and *PLAGL2*-amplified tumors. **b, c** Chromosome 6 and chromosome 20 amplifications in ET, PLAGL tumors were verified using IGV. Samples are grouped according to their amplification status. **b** Shown are focal amplifications on chromosome 6 and chromosome 20 for *PLAGL1* and *PLAGL2*, respectively. **c** Zooming in on the amplified regions around *PLAGL1* and *PLAGL2* (with co-amplification). **d** GISTIC amplification plot of all 31 samples belonging to the ET, PLAGL type. The genome is displayed vertically on the y-axis and genomic positions of amplified regions are indicated. Normalized amplification signals (G-score) and *q* values (log scale) are indicated on the *X*-axis on the top and bottom, respectively. The green line represents the significance cutoff (*q* value = 0.25)

We derived copy number (CN) plots and assessed CN status for all 31 samples based on the raw intensities of the DNA methylation array probes, which revealed focal amplification of *PLAGL1* or *PLAGL2* in 28 of the 31 core samples (90.3%) with 11 samples being *PLAGL1*-amplified (35.5%) and 17 samples being *PLAGL2*-amplified (54.8%). Three samples showed no amplification of any PLAG-family gene (9.7%). CN summary plots were derived for *PLAGL1*- and *PLAGL2*-amplified samples separately to visualize broad chromosomal copy number changes in each subtype (Fig. 2a). As the segmentation algorithm used to produce the summary plots does not always recognize amplicons of very small size as a segment, only a subset of the *PLAGL2* amplifications were detected automatically, but manual screening of the PLAGL-regions confirmed focal amplification of *PLAGL1* or *PLAGL2* as described above (Fig. 2b, c). Differential comparative analysis was performed using GISTIC2.0 to compare *PLAGL1*-amplified versus *PLAGL2*-amplified samples and detect significantly altered regions across all samples and per subtype (Fig. 2d, Supplementary Fig. 3).

Ten of the 17 *PLAGL2*-amplified samples (58.8%) showed co-amplification of a region immediately downstream of *PLAGL2* on chromosome 20, which mainly affected the gene *CBFA2T2* (Fig. 2b, c). GISTIC2.0 analysis confirmed the region containing *PLAGL1* (6q24.2; *q* value 4.83*10^–23^), *PLAGL2* (20q11.21; *q* value 2.20*10^–22^), and the downstream region of co-amplification on 20q11.21 (*q* value 2.98*10^–16^) (Fig. 2d; Supplementary Tables S2, S3) as significantly amplified segments in the *PLAGL1-* and *PLAGL2-*amplified tumors, respectively. Multiple ET, PLAGL group-wide and subgroup-specific deletions were identified, including a common deletion of region 11p15.4 (*q* value 2.07*10^–11^) (Supplementary Fig. 3, Supplementary Tables S2, S4), but this region encompassing various olfactory receptors as well as further affected genomic regions are likely to represent copy number polymorphisms and/or technical artifacts rather than functional somatic alterations.

### Patient and sample characteristics

Patient characteristics were summarized (*n* = 31, Table 1) and visualized (Supplementary Fig. 2). The median age as well as the age range differed between *PLAGL1-* and *PLAGL2-*amplified tumors (p = 0.005497, Wilcoxon rank sum test). *PLAGL1*-amplified cases occurred mainly in school age children and teenagers, with only a few younger patients (1–19 years, median age of 10.5 years), while *PLAGL2*-amplified cases were mostly prevalent in infants and toddlers, with an age range from 1 to 5 years (with the exception of one adult case of 36 years; median age of 2 years). The three ET, PLAGL tumors without *PLAGL1/2* amplification occurred in very young patients (0–2 years, median age of 1). The incidence of *PLAGL1* tumors was higher in girls than in boys (M:F 3:8), while the opposite trend was seen for the incidence of *PLAGL2* tumors (M:F 10:7), but this difference in sex distribution was not statistically significant (p = 0.1021, Chi-square test). Tumors occurred at several anatomic sites, mainly the cerebral hemispheres (35.5%) and the cerebellum (25.8%), but were also found in the brainstem (6.5%), other midline structures (9.7%), or growing into the ventricles (6.5%). Nine out of ten hemispheric PLAGL-amplified tumors occurred in female patients, compared to only three out of seven cerebellar PLAGL-amplified tumors and one tumor that was growing into the ventricles (Supplementary Fig. 2). Initially rendered histopathological diagnoses based on morphological features comprised various tumor types—medulloblastoma (16.1%), other embryonal tumors (22.6%), HGGs (19.4%), indeterminate neuroepithelial tumors (9.7%), and sarcoma (3.2%) (Table 1).

**Table 1:** Patient characteristics for the cohort of CNS embryonal tumors with PLAG-family gene amplification (ET, PLAGL) samples (*n* = 31)

Amplified PLAG-family gene	*PLAGL1*	*PLAGL2*	None	Total
Genomic region	6q24.2	20q11.21	–	–
Patients, *n*	11 (35.5%)	17 (54.8%)	3 (9.7%)	31
*Age, years*				
Median	10.5*	2*	1	–
Range	1–19	0–36	0–2	0–36
*Age group*				
Pediatric	10	14	2	26 (83.9%)
Adult	–	1	–	1 (3.2%)
Unknown	1	2	1	4 (12.9%)
*Sex*				
Female	8^†^	7^†^	2	17 (54.8%)
Male	3^†^	10^†^	1	14 (45.2%)
*Anatomic site*				
Hemispheric	5	5	1	11 (35.5%)
Cerebellum	4	3	1	8 (25.8%)
Infratentorial, midline	–	1	–	1 (3.2%)
Supratentorial, midline	–	2	–	2 (6.5%)
Brainstem	1	1	–	2 (6.5%)
Ventricles	–	2	–	2 (6.5%)
Unknown	1	3	1	5 (16.1%)
*Original histopathologic diagnosis*				
Medulloblastoma	1	3	1	5 (16.1%)
Other embryonal^a^	4	2	–	6 (19.4%)
HGG^b^	1	5	–	6 (19.4%)
NET	1	2	–	3 (9.7%)
Sarcoma	1	1	–	2 (6.5%)
NOS/NEC	3	4	2	9 (29.0%)

Percentages may amount to > 100% due to rounding

HGG, high grade glioma; NET, neuroepithelial tumor; NOS, not otherwise specified; NEC, not elsewhere classified;

*Wilcoxon rank sum test, *α* = 0.05, *p*-value = 0.005497

^†^Chi-square test, *α* = 0.05, *p*-value = 0.1021

^a^Includes: PNET, ETANTR, embryonal tumor

^b^Includes: GBM, glioma

### Targeted next-generation DNA sequencing analysis

A subset of the tumors (*n* = 14) were further analyzed by targeted next-generation DNA sequencing to interrogate the genomic landscape beyond *PLAGL* amplification (Supplementary Table S1). One tumor with *PLAGL2* amplification (#A113—excluded from the core DNA methylation cohort due to QC issues, but with a clear signal for ET, PLAGL), harbored additional focal high-level amplifications of the *MDM4* oncogene on chromosome 1q32.1 and the *MYCN* oncogene on chromosome 2p24.3. In one tumor with *PLAGL1* amplification (#A388), we found separate focal high-level amplification of the *GLI2* oncogene on chromosome 2q14.2, and in another tumor with *PLAGL1* amplification (#A93), we found focal amplification of *MYB* on chromosome 6q23.3. One *PLAGL2*-amplified tumor (#A105) and one *PLAGL1*-amplified tumor (#A93) harbored deleterious missense mutations in the *TP53* tumor suppressor gene. The remaining seven tumors with *PLAGL2* amplification and three tumors with *PLAGL1* amplification harbored no additional likely oncogenic amplifications, homozygous deletions, mutations, insertions/deletions, or gene fusions among any of the evaluated genes. Specifically, all 14 evaluated tumors were wild type for the *IDH1* and *IDH2* genes, as well as the histone H3 genes (*H3F3A*, *H3F3B*, *HIST1H3B*, and *HIST1H3C*). None harbored amplifications, fusions, or mutations of receptor tyrosine kinase genes including *EGFR*, *PDGFRA*, *FGFR1*, *MET*, *ALK*, *ROS1*, or *NTRK2* that are common in pediatric HGG. None harbored alterations within genes of the MAP kinase signaling pathway (e.g., *BRAF*, *KRAS*, *NF1*, and *PTPN11*) that are also common in pediatric gliomas. None harbored mutation or deletion of the *SMARCB1* or *SMARCA4* genes, thereby distinguishing these tumors from atypical teratoid/rhabdoid tumors. None harbored *DICER1* mutation or amplification of the chromosome 19 microRNA cluster (C19MC), thereby distinguishing these tumors from embryonal tumor with multilayered rosettes. None harbored *BCOR* fusions or internal tandem duplication, and none of the evaluated tumors (*n* = 6) harbored *MN1* or *BEND2* fusions. Genetic alterations known to contribute to telomere maintenance (*TERT* promoter mutation or *ATRX* mutation/deletion) were also not identified in any of the tumors.

### Histopathological characterization

**Fig. 3:**
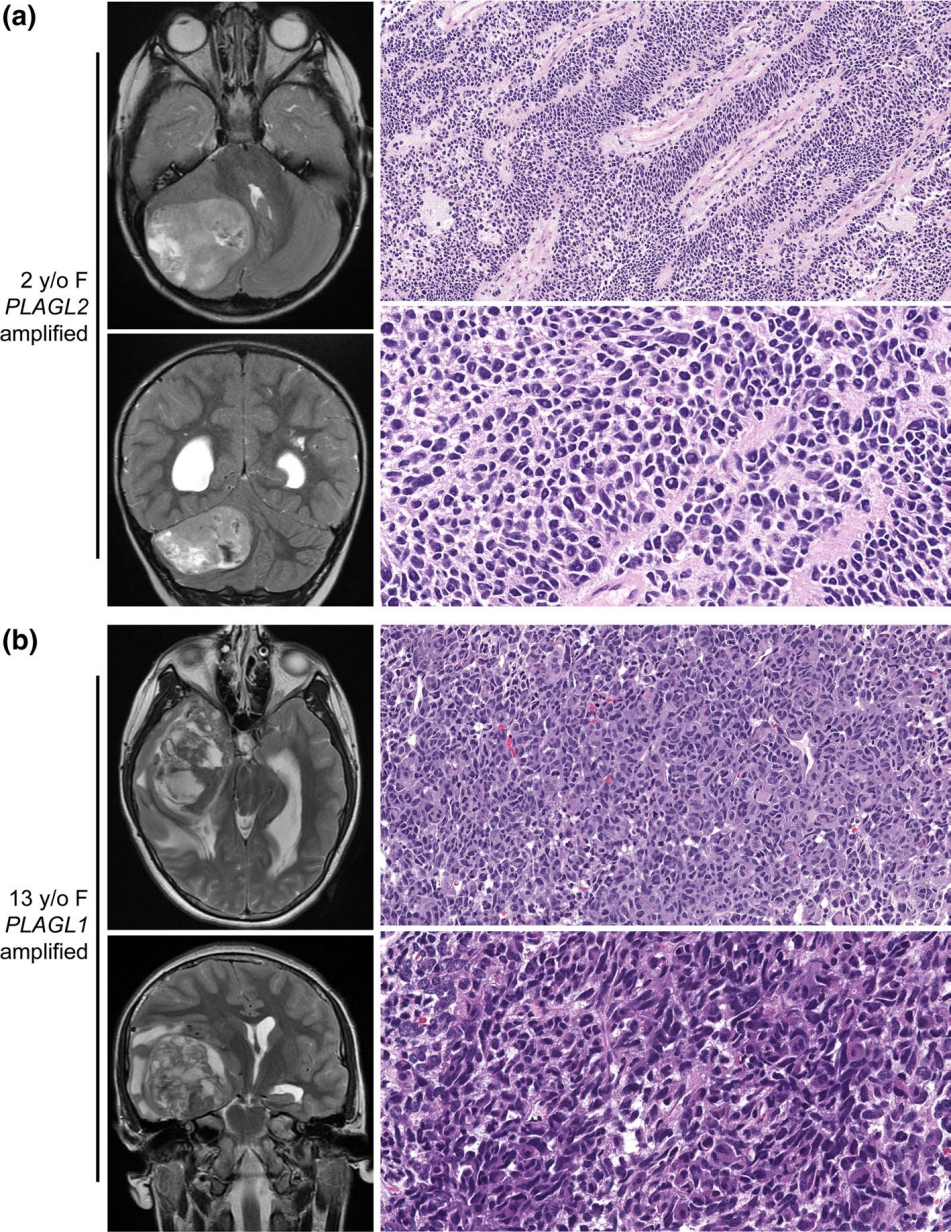
Imaging and histologic features of CNS embryonal tumors with PLAGL gene amplification. Shown are pre-operative T2-weighted MR images and low/high resolution H&E-stained histology images of **a** a *PLAGL2*-amplified tumor in a 2-year-old female patient (#A110) and **b** a *PLAGL1*-amplified tumor in a 13-year-old female patient (#A387)

Histopathological review was performed on a subset of the tumors, including 6 with *PLAGL2* amplification, 8 with *PLAGL1* amplification, and 1 with no PLAG gene family amplification. The predominant morphological pattern was a densely cellular neoplasm with solid growth composed of primitive, embryonal-like cells with brisk mitotic activity (Fig. 3, Supplementary Fig. 4, and Supplementary Table S5). Less common patterns included spindled and more uniform/monotonous round cells, but tumors with these patterns always had other areas with more primitive, embryonal-like cells. While most tumors demonstrated a solid growth pattern with a paucity of entrapped neuropil and a sharply circumscribed border with adjacent brain parenchyma, a couple of tumors displayed focal infiltrative growth. Many tumors had regions of necrosis, usually without palisading of tumor cells at the periphery. No well-developed microvascular proliferation was observed in any of the reviewed tumors. Ependymal canals or perivascular pseudorosettes (characteristic histological features of ependymoma) were not observed.

**Fig. 4:**
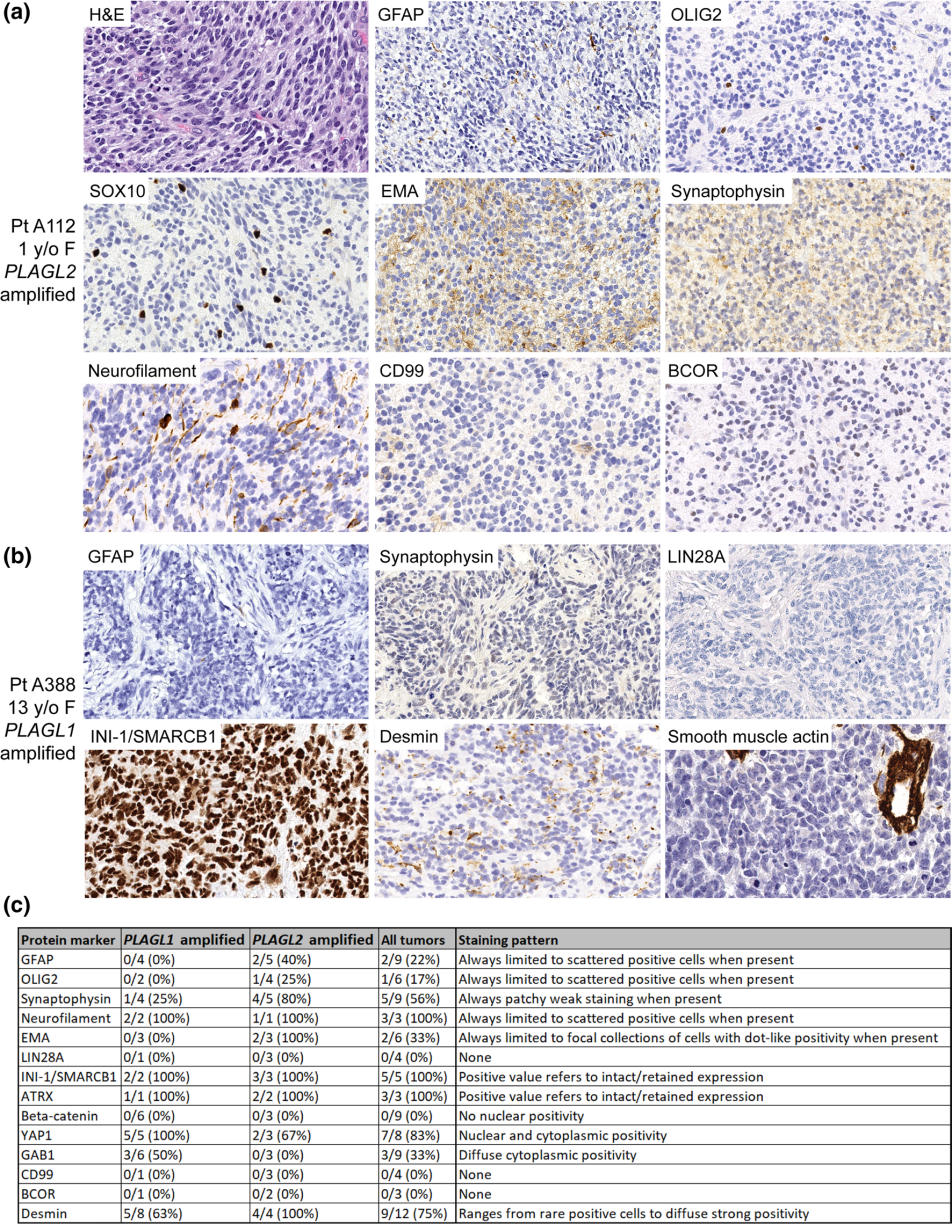
Immunohistochemical features of CNS embryonal tumors with PLAGL gene amplification. Shown are representative immunostains of **a** a *PLAGL2*-amplified tumor in a 1-year-old female patient and** b** a *PLAGL1*-amplified tumor in a 13-year-old female patient. **c** Summary of IHC results in *PLAGL1/2*-amplified tumor samples

Immunostaining for markers of glial differentiation (GFAP and OLIG2) was mostly negative, with only a few *PLAGL2*-amplified tumors showing labeling of rare scattered tumor cells (Fig. 4, Supplementary Table S5). Several tumors demonstrated patchy weak staining for synaptophysin, while others were negative. Neurofilament expression was often seen in scattered tumor cells, but otherwise revealed an absence of entrapped neuropil, confirming the solid growth pattern of these tumors. Two *PLAGL2*-amplified tumors demonstrated focal collections of tumor cells with paranuclear dot-like positivity for EMA staining, while the majority of tumors lacked EMA expression. All evaluated tumors had intact/retained expression of INI1/SMARCB1 and BRG1/SMARCA4. All evaluated tumors had minimal to absent immunostaining for LIN28A, BCOR, and CD99. A subset of tumors demonstrated positivity for YAP1 and GAB1, while no tumors had nuclear beta-catenin staining. Desmin expression was present in the majority of evaluated tumors (9/12, 75%), which ranged from rare scattered cells to diffuse strong labeling of all tumor cells in a small number of the *PLAGL2*-amplified cases. Other markers of myogenic differentiation (myogenin, smooth muscle actin, and MyoD1) were negative in all evaluated tumors. Ki-67 labeling indices ranged from 30 to 70%.

### Gene expression analysis

**Fig. 5:**
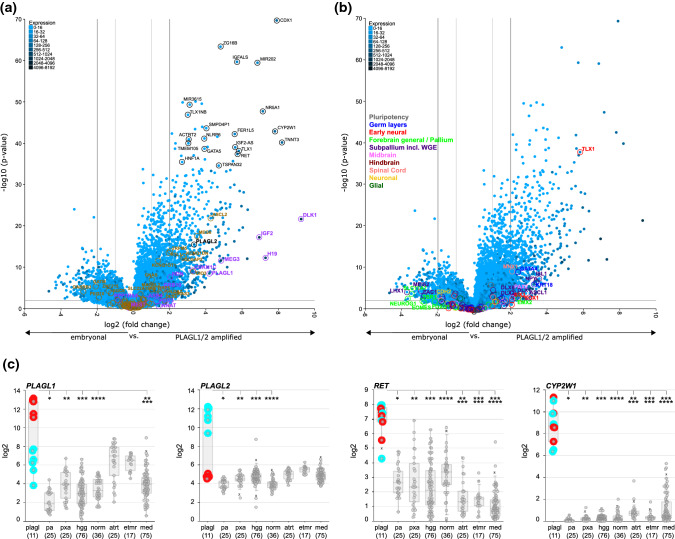
Gene expression profiles of CNS embryonal tumors with PLAGL gene amplification. **a**, **b** Volcano plots showing fold-change and p-value for the comparison of differential gene expression of 11 *PLAGL1*/*2*-amplified tumors versus 117 embryonal tumors from different types and subtypes. Highlighted are **a** 86 human IGs (ocher) and 13 IGs with high connectivity (lilac) as described in reference [5]. Shown in black: selection of genes with large magnitude fold-changes (*x* axis) and high statistical significance (− log10 of *p*-value, *y*-axis). **b** Genes with differential expression in different brain regions and during different developmental states as described in reference [56] **c** Boxplots comparing gene expression between CNS tumor types for a select set of genes. The subset of 117 embryonal tumor samples (atrt, etmr, med) is identical to **a** and **b**. plagl, ET,PLAGL; pa, pilocytic astrocytoma; pxa, pleomorphic xanthoastrocytoma; hgg, high-grade gliomas (G34R/V, K27M, pedRTK1/2); norm, normal brain tissues; atrt, atypical teratoid rhabdoid tumor; etmr, embryonal tumor with multilayered rosettes; med, medulloblastomas (WNT, SHH, group 3, group 4); red: samples with *PLAGL1* amplification, blue: samples with *PLAGL2* amplification. Significance bars indicate groups whose differences in gene expression are statistically significant when compared to samples with *PLAGL1/2* amplification (*t*-test, Bonferroni-corrected *p*-value = 0.00714286). PLAGL1/2 upregulation is statistically significant compared to all other groups when looking at PLAGL1 or PLAGL2 tumors separately

Differential gene expression between tumors with PLAG-family gene amplification and a selection of other CNS tumor types was examined using the R2 Genomics Analysis and Visualization Platform (http://r2.amc.nl). In concordance with the observed gene amplification, ET, PLAGL tumors showed overexpression of the respective amplified PLAG-family gene as assessed by RNA-seq (Fig. 5a, c), while both PLAGL1 and PLAGL2 are downregulated postnatally in normal brain and cerebellar tissues (https://apps.kaessmannlab.org/evodevoapp/) [13] (Supplementary Fig. 5). Leveraging our expression data set of 11 *PLAGL1*- and *PLAGL2*-amplified tumor samples and 279 samples from other CNS tumor and normal tissue types (HGGs with H3 G34R/V or K27M mutation and GBM_pedRTK1 or 2 (*n* = 76), PA with *BRAF* fusion (*n* = 25), PXA (*n* = 25), normal brain tissue (*n* = 36), embryonal tumors such as ATRT, ETMR, or medulloblastomas (*n* = 117)), we first compared gene expression of the PLAGL-amplified tumors to our subset of embryonal tumors. We derived a gene set specific to the ET, PLAGL type (Fig. 5a) as well as a PLAGL-specific gene-signature consisting of the top 250 differentially expressed genes (Supplementary Table S6). In addition to PLAGL1/2 overexpression, we found differential expression of several genes involved in developmental and differentiation processes such as *CDX1*, *NR5A1*, *TLX1*, *TBX1*, *FGF19*, and *DLK1* (Fig. 5a, Supplementary Table S6); known direct *PLAGL* target genes such as *IGF2*, *H19*, *CDKN1C* and *DLK1* [64] (Fig. 5a, Supplementary Fig. 6), as well as *CYP2W1* and the kinase *RET*, both putative treatment targets (Fig. 5a, c). We screened expression of 86 human IGs in the *PLAGL1/2*-amplified samples (Fig. 5a). A subset of 13 IGs (*Meg3*, *Ndn*, *Grb10*, *Dlk1*, *Igf2*, *Cdkn1c*, *Plagl1*, *Peg3*, *Mest*, *Nnat*, *Asb4*, *H19*, and *Ppp1r9a*) described as having high connectivity with other IGs [5] were differentially expressed in the *PLAGL1/2*-amplified tumors (Fig. 5a, Supplementary Figs. 6, 7). We also ran the same differential expression analyses comparing ET, PLAGL versus glial tumors as well as versus normal fetal and adult brain tissues. This analysis yielded similar results regarding the overrepresentation of imprinted genes as well as developmental and differentiation-related genes (Supplementary Fig. 8). Expression of classical pan-neuronal, glial, sarcoma/mesenchymal, neural stem cell, and proliferation marker genes was also examined in the ET, PLAGL tumors versus our subset of CNS embryonal tumors, gliomas, and normal tissues, but was inconclusive in terms of possible cell/lineage of origin, as there was no set of marker genes that was clearly differentially expressed in the ET, PLAGL tumor type—with the exception of high *Desmin* expression in the PLAGL-amplified tumors in all three comparisons (Supplementary Fig. 9). Overexpression of the myogenic marker *Desmin* was more pronounced in the *PLAGL2*-amplified samples (Supplementary Fig. 9d). Furthermore, this analysis showed a lack of glial marker expression in the PLAGL tumors. We compared bulk RNA-seq data of the ET, PLAGL tumors to a single-nucleus sequencing atlas containing transcriptomes from different cell types, differentiation states, and subtypes of the developing human cerebellum to map the cellular origins of the ET, PLAGL tumors (Supplementary Fig. 10) as described in Okonechnikov et al. [39]. None of the lineages that were used as reference could be identified as the origins of ET, PLAGL tumors. Consequently, we analyzed the expression of genes representing different developmental states and locations as markers for pluripotency, germ layers (ectoderm, mesoderm, endoderm), neuroectoderm, forebrain and pallium, subpallium (including the ganglionic eminence), midbrain, hindbrain, spinal cord, as well as various additional pan-neuronal and glial markers [56] (Fig. 5b, Supplementary Fig. 11). The early neural genes *OTX2*, *TLX1*, *SIX3*, *MSI1*, and *DACH1* were overexpressed in the PLAGL-amplified tumors, as were some subpallial neural markers such as *DLX5*, *DLX6*, the lateral ganglionic eminence (LGE) marker *ISL1*, and the germ layer markers *KRT18* and *GATA4* pointing to a cell of origin at an early and rather undifferentiated developmental stage.

### ChIP-seq analysis

Chromatin immunoprecipitation studies using antibodies against PLAGL1 and PLAGL2 proteins (*n* = 5) was performed to identify gene loci bound by these two transcription factors in PLAGL-amplified tumors. This ChIP-seq data confirmed the known *Plagl1* targets *IGF2*, *CDKN1C,* and *DLK1*, as well as most of the other IGs with high connectivity, as being direct targets of *PLAGL1* and *PLAGL2* TF binding in the PLAGL-amplified tumors (Supplementary Fig. 6, Supplementary Fig. 12). The receptor tyrosine kinase *RET* and the cytochrome P450 family member *CYP2W1*—both potential drug targets—were also revealed as further direct *PLAGL1/2* targets (Supplementary Fig. 13). In addition, we identified components of the Wnt/β-Catenin signaling pathway, *FZD2* and *FZD9*, to be targets of *PLAGL1/2* TF binding that are also differentially expressed in the PLAGL-amplified tumors (Supplementary Fig. 14).

### Survival analysis

**Fig. 6:**
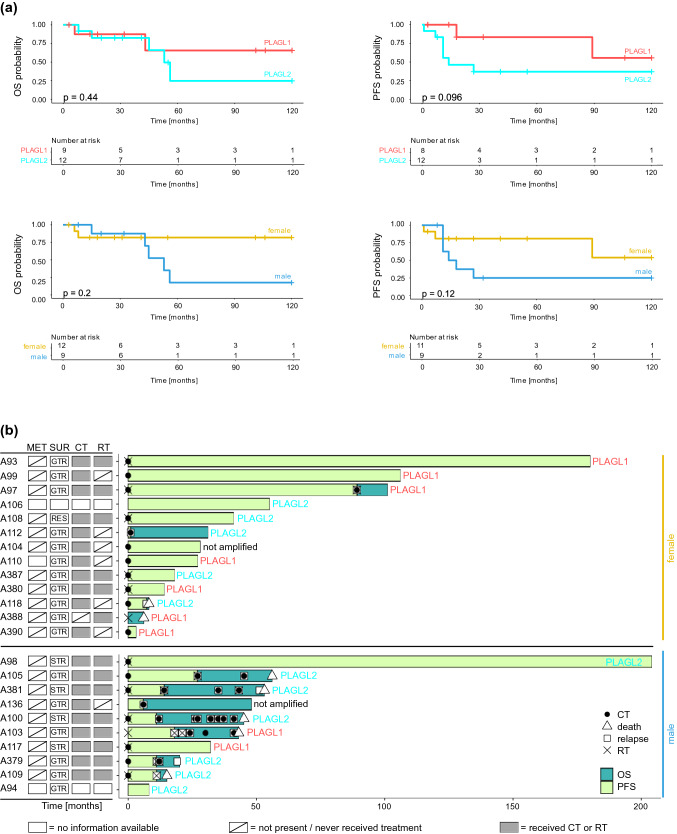
Clinical outcomes of patients with CNS embryonal tumor with PLAGL gene amplification. **a** Kaplan–Meier plots showing OS and PFS stratified by subgroup and sex. The log-rank test was used to show differences between the curves, p-values of the log-rank test are shown in each graph. **b** Swimmer plot showing available OS and PFS times per patient, including treatment information and clinical response/relapse. Samples are stratified by sex, *PLAGL1/2* amplification status is indicated. Information about surgical resection (SUR) and presence of metastasis (MET) at the time point of primary diagnosis is displayed in the squares on the left where available (resections or metastases at later time points are not displayed), GTR, gross total resection; STR, subtotal resection; RES, resection (unknown, if GTR or STR). Information about chemotherapy (CT) and radiotherapy (RT) treatment regarding the entire follow-up time is displayed in the squares on the left where available

Clinical outcome data were available for 21 patients with *PLAGL1/2*-amplified tumors. Five-year and 10-year OS for patients with *PLAGL1*- and *PLAGL2*-amplified tumors as well as for male and female patients was determined. Survival rates across the cohort remained constant after 5 years, hence both 5- and 10-year OS was 66% for patients with *PLAGL1*-amplified tumors, 25% for patients with *PLAGL2*-amplified tumors, 18% for male patients, and 82% for female patients, respectively. Although a trend towards a worse prognosis for patients with *PLAGL2*-amplified tumors was noticeable—with 5 out of 12 patients with a *PLAGL2*-amplified tumor being deceased compared to 2 out of 9 patients with a *PLAGL1*-amplified tumor (Fig. 6b)—PFS and OS did not differ significantly between the two different groups (Fig. 6a, *p* value = 0.096 and 0.44, respectively). Patient sex was also not a significant predictor for PFS or OS (Fig. 6a, *p* value = 0.12 and 0.2), but more deaths in male patients were recorded irrespective of the subgroup. With respect to different treatment regimens, the inclusion of chemotherapy agents beyond temozolomide (TMZ) early on in treatment showed a potential benefit for patient survival (Fig. 6b, Supplementary Table S7) while the inclusion of radiotherapy as part of the initial treatment seemed to have limited effect (Supplementary Fig. 15), but this should be judged with caution given the overall low numbers.

## Discussion

We describe a rare, novel type of pediatric CNS tumor with a distinct methylation pattern that we name “CNS embryonal tumor with PLAGL amplification” (abbreviated as ET, PLAGL). The described new type is epigenetically divergent from all other described CNS tumor types and in the vast majority of cases marked by amplification of one of the PLAG-family genes, *PLAGL1* or *PLAGL2*. Despite the initial diagnosis as medulloblastoma, other embryonal tumor, or HGG in more than half of cases, PLAGL-amplified tumors differ both epigenetically and in terms of outcome from these other tumor types. Initial histopathologic diagnoses were variable, and many tumors had been considered not classifiable despite multiple differential diagnoses being discussed. t-SNE dimensionality reduction of DNA methylation profiles unraveled further substructure within the PLAGL group in accordance with the respective gene amplification—*PLAGL1* or *PLAGL2*. A subset of the *PLAGL2*-amplified cases also harbored co-amplification of a downstream region on chromosome 20 as possible evidence for more complex structural rearrangements, which will be of interest to explore further in larger tumor cohorts.

*PLAGL1*, *PLAGL2*, and also *PLAG1* belong to the PLAG gene family [20]. *PLAG1* and *PLAGL2* have been suggested to be proto-oncogenes with comparable DNA-binding capacities and partly overlapping functions [4, 20]. *PLAG1* promoter swapping and subsequent activation is reported as playing a key role in the development of pleomorphic adenomas of the salivary gland, lipoblastomas, hepatoblastomas, and some leukemias, and *PLAG1* overexpression has been found in uterine leiomyomas, leiomyosarcomas, and other smooth muscle tumors [4, 65, 66]. *PLAGL2* has been found to be amplified in a small subset of cancers, and has been reported to promote tumorigenesis together with *POFUT1* in colorectal cancer [4, 32]. Overexpression of *PLAGL2* has been reported to play a role in lung adenocarcinoma development, and may represent a poor prognostic marker in prostate cancer [18, 69]. The role of *PLAGL1* is less clear, and it may potentially act as both tumor suppressor and oncogene depending on the context. *PLAGL1* was first discovered as a zinc finger protein that concurrently induces apoptosis and cell cycle arrest [55]. The maternally imprinted *PLAGL1* is expressed in normal tissues and downregulated in various tumors, such as breast cancer, ovarian cancer, nonfunctioning pituitary tumors, basal cell carcinomas, head and neck squamous cell carcinoma, and diffuse large B-cell lymphoma [3, 8, 30, 64]. It was shown to downregulate proliferation through induction of PPARɣ in human colon carcinoma cells [8]. In contrast to these findings, overexpression of *PLAGL1* was found to contribute to the tumorigenesis of glioma-initiating cells [21] and recurrent *PLAGL1* fusions (most commonly with *EWSR1*) were found to characterize a novel subtype of supratentorial ependymoma-like tumor in pediatric patients [54]. Our findings further support that in a brain tumor context, both *PLAGL1* and *PLAGL2* likely act as oncogenes, whose amplification and resultant overexpression drive tumor development.

In addition to the apparent epigenetic differences between *PLAGL1*- and *PLAGL2*-amplified tumors discovered through t-SNE analysis, we noted further distinctions between the two subtypes. *PLAGL1*-amplified tumors rarely occurred in young children and were more prevalent in school-aged children and teenagers, while *PLAGL2*-amplified cases were mostly prevalent in infants and toddlers. While *PLAGL1* is ubiquitously expressed in many normal fetal and adult tissues, *PLAGL2* is only expressed in fetal tissues [2]. Accordingly, the median age at diagnosis was significantly lower for the *PLAGL2*-amplified tumors than for the *PLAGL1*-amplified tumors. Although not significant, a trend towards a more favorable clinical outcome in *PLAGL1*-amplified tumors was noted. One possibility is that amplification of *PLAGL2* leading to tumor formation can only occur during a small spatiotemporal window during development when *PLAGL2* is expressed, while amplification of *PLAGL1* might be less temporally limited in terms of subsequent tumor formation. Alternatively, considering the less aggressive phenotype and more favorable outcome, *PLAGL1* activation might lead to slower growing tumors that only become symptomatic in older children, even if they arise around the same time during development and target the same cell type of origin.

We further report clinical differences between male and female patients within this tumor type. The incidence of *PLAGL1* tumors was higher in female patients, while the incidence of *PLAGL2* tumors was higher in male patients. In terms of location, cerebral hemispheric tumors were more prevalent in females, while cerebellar, brainstem, and other midline tumors were more prevalent in males. Sex-specific differences were also noted in terms of outcome, with female patients showing a trend towards more favorable survival. Since the patients who succumbed to their disease more often had tumors in the cerebellum or midline structures, one possible explanation apart from molecular differences between the tumors might be the more surgically accessible tumor location in the cerebral hemispheres, which is predominantly found in female patients and might better enable total surgical excision. Outcomes do not seem to be related to differences in the specific PLAGL gene that is amplified or treatment between the two sexes based on the patient cohort to date, although it should be noted that there was some evidence of improved outcomes for early chemotherapeutic intervention for incompletely resected tumors.

Gene expression profiles of 11 tumors with amplification of *PLAGL1* (*n* = 5) or *PLAGL2* (*n* = 6) were compared to gene expression profiles of a total of 279 tumor samples from various other CNS tumor types and normal tissues. Overexpression of a subset of 13 IGs—*MEG3*, *NDN*, *GRB10*, *DLK1*, *IGF2*, *CDKN1C*, *PLAGL1*, *PEG3*, *MEST*, *NNAT*, *ASB4*, *H19*, *PPP1R9A—*was specific for the PLAGL-amplified tumors. These 13 genes are imprinted in humans and are reported to have high connectivity with other genes belonging to the IGN in mouse [5]. Plagl1 was previously shown to regulate expression of *Cdkn1c*, *Igf2*, *H19,* and *Dlk1* and to belong to a subset of IGs that control embryonic growth and differentiation, and loss of Plagl1 function resulted in intrauterine growth restriction [64]. We show through analysis of ChIP-seq data that *PLAGL1* and *PLAGL2* bind directly upstream of the majority of the 13 IGs in human *PLAGL1/2*-amplified tumors, which underlines similarities in the mouse and human networks. Furthermore, 9 of the 13 genes—*Igf2*, *H19*, *Plagl1*, *Mest*, *Peg3*, *Dlk1*, *Grb10*, *Ndn*, *Cdkn1c*—were reported to belong to a subset of 11 IGs which were downregulated postnatally in an age-dependent fashion accompanying a decline in growth rate [36]. The same set of nine genes was found differentially expressed in different types of mouse and human somatic stem cells compared to their differentiated counterparts, and the expression of those IGs correlated with stem cell properties [9]. A subset (*MEST*, *PLAGL1*, *PEG3*, *DLK1*, *IGF2*) showed elevated expression in various embryonal cancers, especially rhabdomyosarcoma, as compared to non-embryonal cancers and normal tissues. The whole set of nine genes was found overexpressed in mouse embryoid bodies—aggregates of embryonic stem cells (ESCs) undergoing differentiation and comprising differentiated cell phenotypes of all three germ lineages [10, 29]—but not in ESCs [9, 44]. Further literature confirms the role of *PLAGL1* as a “master switch”, a TF that regulates a substantial fraction of the IGN genes and extracellular matrix (ECM) genes, and may regulate a subset of neuroblastoma signature genes [63].

IGs are considered to be key regulators of embryonic development and global loss of imprinting (LOI) as well as LOI of *IGF2*, which is regulated by *PLAGL1*, can lead to tumor formation [5, 22, 64]. It was also shown that overexpression of *Plagl1* abolished the neuronal commitment of non-glioma-initiating cells and caused them to become malignant [21]. Therefore, we conclude that *PLAGL1* amplification and subsequent overexpression may contribute to tumor formation depending on the cell of origin and developmental state. The above-named subset of 13 IGs was also overexpressed to the same extent in *PLAGL2*-amplified samples, despite the fact that *PLAGL2* is not an IG in humans and has not been associated with the mouse IGN of 409 (85 imprinted and 324 non-imprinted) genes published by Al Adhami et al. [5]. However, significant overlap was found between *Plagl1* target genes and *PLAG1* and *Plagl2* targets [63]. *PLAGL2* was shown to upregulate IGF2 expression levels in hematopoietic progenitors of acute myeloid leukemia and *IGF2* harbors eight PLAG1/PLAGL2 consensus binding sites [31]. *PLAGL2* was also reported to activate the IGF2 signaling pathway in colorectal cancer [34]. It is, therefore, possible that amplification and overexpression of *PLAGL2* as well as subsequent upregulation of IGF2 in turn lead to both upregulation of genes of the IGN, specifically of those 13 genes with high connectivity, and activation of the IGF mitotic signaling pathway. Amplification and subsequent overexpression of *PLAGL2* was also shown to suppress differentiation in neural stem cells and glioma-initiating cells, in part through aberrant Wnt/β-Catenin signaling in malignant glioma [71]. In keeping herewith, we show through ChIP-seq data that components of the Wnt-pathway such as *FZD2* and *FZD9* are direct targets of PLAGL1 and PLAGL2 and are overexpressed in the *PLAGL1/2*-amplified samples compared to the other CNS tumor types. Further genes that were found to be overexpressed as well as direct PLAGL1/2 targets are the receptor tyrosine kinase *rearranged* *during* *transfection* (*RET*) and the cytochrome P450 family gene *CYP2W1*. *RET* plays a role in the development of the nervous system, where it is expressed in neural crest cells. It was reported to be oncogenic through gene rearrangements, activating mutations, or overexpression of the wild-type gene in multiple cancers, and various small molecule inhibitors targeting *RET* are available or being tested in clinical trials [15, 51, 57]. *CYP2W1* belongs to the cytochrome P450 superfamily of monooxygenases that are involved in xenobiotic metabolism [53]. *CYP2W1* was found to be selectively expressed in colon cancer tissues, but not in healthy tissues making it a putative tumor-specific drug target [61]. The fact that it was also found highly overexpressed in our cohort of *PLAGL*-amplified tumors indicates its potential for targeted treatment, for example, with seco-duocarmycin based prodrugs that are converted into cytotoxic metabolites upon bioactivation by *CYP2W1*, or with antibody drug conjugates (ADC) whose payload gets released upon antibody cleavage [7, 49, 70].

Since our intent to map the cellular origins using a single-nucleus atlas of the developing human cerebellum did not yield informative results, we screened the expression of classical marker genes as well as multiple genes with differential expression in various regions and developmental states of the brain to investigate the potential underlying cell of origin of *PLAGL*-amplified CNS tumors. Glial, astroglial, and oligodendrocytic markers were clearly under-expressed compared to glial tumors and normal brain tissues, making it unlikely that PLAGL-amplified tumors are of purely glial origin. The neural stem cell markers *SOX2* and *Nestin* were also either not differentially expressed or under-expressed in the *PLAGL*-amplified type compared to the other CNS tumor types. Neuronal marker expression did not unambiguously point to a neuronal tumor, as classical neuronal markers were not over-represented among the differentially expressed genes specific to the *PLAGL*-amplified tumors. The germ layer markers *GATA4* and *KRT18* were over-represented in the ET, PLAGL tumors and concurrently, some neuronal genes were also expressed to some extent—*TUBB3*, which is expressed in neuronal restricted progenitor cells [47], and several early neural genes [56] such as TLX1, which is a key TF in the determination of neuronal cell fates [19, 45], were overexpressed in the PLAGL type, potentially suggesting a neuronal lineage at an early developmental state. During mouse brain development (E12-E14—corresponds to 36–48 days post conception in humans) the mouse orthologue *Plagl1* shows strong expression in the neural tube and several neuroepithelia such as the telencephalic vesicles (forebrain region) as well as the third (midbrain) and fourth (hindbrain) ventricles. *Plagl1*-expressing cells are further found in the spinal cord, co-localized with *Tubb3* in the outer cell layer of the subventricular zone of the neuroepithelia, in the brainstem and many more proliferative areas [62], which is consistent with our finding of high expression of early neural genes as well as marker genes localized in the forebrain, midbrain, hindbrain, or spinal cord and could also be an explanation for the diverse tumor sites we report. We found overexpression and differential expression of several genes that are highly expressed in the human ganglionic eminence (GE) at around 7–9 p.c.w.—*DLX5*, *DLX6,* and the aforementioned *SIX3*—as well as *DLX2,* which is expressed before 7 weeks p.c. and *ISL1*, a neuroblast marker expressed in the lateral ganglionic eminence (LGE) around 8 weeks p.c. [24, 56]. In general, all three PLAG-family genes are found at higher levels in neural progenitor cells than in post-mitotic neurons [4]. Data derived from scRNA-seq from the first trimester of human development showed a gradual transition from neuroepithelial to radial glia populations rather than specific gene programs that distinguish progenitor groups—Eze et al. describe a *DLK1* overexpressing cortical progenitor cluster, where *DLK1* in combination with low *SOX2* levels, a combination we also see in the PLAGL-amplified tumors, was highly enriched in early samples and disappeared after CS16 [17]. In addition, we find concurrent expression of *BCL11B* and to some extent *DCX* in the PLAGL-amplified tumors—which were described as markers of maturing and newborn neurons, respectively. Overall, these data support that *PLAGL1/2*-amplified tumors may arise from early neural progenitor cells with possible early commitment to a neuronal lineage. However, the morphology and other histological features confirm only an undifferentiated, embryonal-like pattern. We, therefore, propose the name ET, PLAGL, for CNS embryonal tumor with PLAGL gene amplification.

## Conclusion

We describe a novel, biologically distinct type of CNS embryonal tumor—ET, PLAGL—that is presumably driven by amplification and subsequent overexpression of *PLAGL1* or *PLAGL2*, the only recurrent molecular event detected in these tumors. Dysregulation of imprinted genes is a common feature in the *PLAGL1/2*-amplified tumors that might contribute to tumor formation. Histopathological appearance is that of a primitive, embryonal-like neoplasm without specific morphologic features or routine immunohistochemical markers that enable the accurate distinguishment of ET, PLAGL from other CNS tumor types. Further studies will be needed to identify ET, PLAGL-specific biomarkers that can be used in routine neuropathology diagnostic practice. Finally, the oncogenic *RET* kinase as well as *CYP2W1* are direct *PLAGL1/2* targets and may be potential drug targets in this tumor type, which should be top priorities for future functional validation.

## Supplementary Information

Below is the link to the electronic supplementary material.

Supplementary file1 (XLSX 5109 KB)

Supplementary file2 (PDF 35368 KB)
