# Correction to: Amplification of the PLAG-family genes—*PLAGL1* and *PLAGL2*—is a key feature of the novel tumor type *CNS embryonal tumor with PLAGL amplification*

**DOI:** 10.1007/s00401-023-02538-4

**Published:** 2023-02-14

**Authors:** Michaela-Kristina Keck, Martin Sill, Andrea Wittmann, Piyush Joshi, Damian Stichel, Pengbo Beck, Konstantin Okonechnikow, Philipp Sievers, Annika K. Wefers, Federico Roncaroli, Shivaram Avula, Martin G. McCabe, James T. Hayden, Pieter Wesseling, Ingrid Øra, Monica Nistér, Mariëtte E. G. Kranendonk, Bastiaan B. J. Tops, Michal Zapotocky, Josef Zamecnik, Alexandre Vasiljevic, Tanguy Fenouil, David Meyronet, Katja von Hoff, Ulrich Schüller, Hugues Loiseau, Dominique Figarella-Branger, Christof M. Kramm, Dominik Sturm, David Scheie, Tuomas Rauramaa, Jouni Pesola, Johannes Gojo, Christine Haberler, Sebastian Brandner, Tom Jacques, Alexandra Sexton Oates, Richard Saffery, Ewa Koscielniak, Suzanne J. Baker, Stephen Yip, Matija Snuderl, Nasir Ud Din, David Samuel, Kathrin Schramm, Mirjam Blattner-Johnson, Florian Selt, Jonas Ecker, Till Milde, Andreas von Deimling, Andrey Korshunov, Arie Perry, Stefan M. Pfister, Felix Sahm, David A. Solomon, David T. W. Jones

**Affiliations:** 1 Hopp Children’s Cancer Center Heidelberg (KiTZ), Im Neuenheimer Feld 280, 69120 Heidelberg, Germany; 2 Division of Pediatric Glioma Research (B360), German Cancer Research Center (DKFZ), Im Neuenheimer Feld 280, 69120 Heidelberg, Germany; 3 Division of Pediatric Neurooncology, German Cancer Consortium (DKTK), German Cancer Research Center (DKFZ), Heidelberg, Germany; 4 Department of Neuropathology, Institute of Pathology, University Hospital Heidelberg, Heidelberg, Germany; 5 Clinical Cooperation Unit Neuropathology, German Consortium for Translational Cancer Research (DKTK), German Cancer Research Center (DKFZ), Heidelberg, Germany; 6 Faculty of Biosciences, Heidelberg University, Heidelberg, Germany; 7 Institute of Neuropathology, University Medical Center Hamburg-Eppendorf, Hamburg, Germany; 8 Geoffrey Jefferson Brain Research Centre, Division of Neuroscience and Experimental Psychology, Faculty of Biology, Medicine and Health, University of Manchester, Manchester, UK; 9 Department of Radiology, Alder Hey Children’s NHS Foundation Trust, Liverpool, UK; 10 Division of Cancer Sciences, University of Manchester, Manchester Academic Health Science Centre, Manchester, UK; 11 Department of Pediatric Hematology and Oncology, Alder Hey Children’s NHS Foundation Trust, Liverpool, UK; 12 Princess Máxima Center for Pediatric Oncology, Utrecht, The Netherlands; 13 Department of Pathology, Amsterdam University Medical Centers, Location VUmc and Brain Tumor Center Amsterdam, Amsterdam, The Netherlands; 14 Department of Pediatric Oncology and Hematology, Skåne University Hospital, Lund University, Lund, Sweden; 15 Department of Oncology-Pathology, Karolinska Institutet, Stockholm, Sweden; 16 Prague Brain Tumor Research Group, Second Faculty of Medicine, Charles University and University Hospital Motol, Prague, Czech Republic; 17 Department of Pediatric Haematology and Oncology, Second Faculty of Medicine, Charles University and University Hospital Motol, Prague, Czech Republic; 18 Department of Pathology and Molecular Medicine, Second Faculty of Medicine, Charles University and University Hospital Motol, Prague, Czech Republic; 19 Institut de Pathologie Multisite-Site Est, Groupement Hospitalier Est, Hospices Civils de Lyon, Lyon, France; 20 Department of Pediatric Oncology and Hematology, Charité-Universitätsmedizin Berlin, Corporate Member of Freie Universität Berlin, Humboldt-Universität zu Berlin, and Berlin Institute of Health, Berlin, Germany; 21 Department of Pediatric Hematology and Oncology, University Medical Center Hamburg-Eppendorf, Hamburg, Germany; 22 Research Institute Children’s Cancer Center Hamburg, Hamburg, Germany; 23 University of Bordeaux, Bordeaux Institute of Oncology (BRIC)-INSERM U1312 Université de Bordeaux, 146 rue Leo Saignat, Case 76, 33076 Bordeaux, France; 24 Aix-Marseille Univ, APHM, CNRS, INP, Inst Neurophysiopathol, CHU Timone, Service d’Anatomie Pathologique et de Neuropathologie, Marseille, France; 25 Division of Pediatric Hematology and Oncology, University Medical Center Göttingen, Göttingen, Germany; 26 Department of Pediatric Oncology, Hematology, Immunology and Pulmonology, University Hospital Heidelberg, Heidelberg, Germany; 27 Department of Pathology, Rigshospitalet, Copenhagen, Denmark; 28 Department of Clinical Pathology, Kuopio University Hospital and Unit of Pathology, Institute of Clinical Medicine, University of Eastern Finland, Kuopio, Finland; 29 Department of Pediatrics, Pediatric Hematology and Oncology Ward, Kuopio University Hospital and Institute of Clinical Medicine, University of Eastern Finland, Kuopio, Finland; 30 Department of Pediatrics and Adolescent Medicine, Comprehensive Cancer Center and Comprehensive Center for Pediatrics, Medical University of Vienna, 1090 Vienna, Austria; 31 Division of Neuropathology and Neurochemistry, Department of Neurology, Medical University of Vienna, Vienna, Austria; 32 Division of Neuropathology, National Hospital for Neurology and Neurosurgery, University College London Hospitals NHS Foundation Trust, Queen Square, London, UK; 33 Department of Neurodegenerative Disease, UCL Queen Square Institute of Neurology, Queen Square, London, UK; 34 Department of Developmental Biology and Cancer, UCL GOS Institute of Child Health, University College London, London, UK; 35 Murdoch Children’s Research Institute and Department of Paediatrics, University of Melbourne, Royal Children’s Hospital, Melbourne, Australia; 36 Department of Pediatric Oncology/Hematology/Immunology, Olgahospital, Klinikum Stuttgart, Stuttgart, Germany; 37 Department of Developmental Neurobiology, St. Jude Children’s Research Hospital, Memphis, TN USA; 38 Department of Pathology and Laboratory Medicine, The University of British Colombia, Vancouver, Canada; 39 Department of Pathology, NYU Langone Medical Center, New York, NY USA; 40 Department of Pathology and Laboratory Medicine, The Aga Khan University, Karachi, Pakistan; 41 Department of Pediatric Hematology-Oncology, Valley Children’s Hospital, Madera, CA USA; 42 Clinical Cooperation Unit Pediatric Oncology, German Consortium for Translational Cancer Research (DKTK), German Cancer Research Center (DKFZ), Heidelberg, Germany; 43 KiTZ Clinical Trial Unit (ZIPO), Department of Pediatric Hematology and Oncology, Heidelberg University Hospital, Heidelberg, Germany; 44 Division of Neuropathology, Department of Pathology, University of California San Francisco (UCSF), 513 Parnassus Ave, Health Sciences West 451, San Francisco, CA 94143 USA

**Correction to: Acta Neuropathologica (2023) 145:49–69** 10.1007/s00401-022-02516-2

In the original publication, incorrect version of Fig. 6 was published and the correct version (Fig. 6) is given below.

**Fig. 6:**
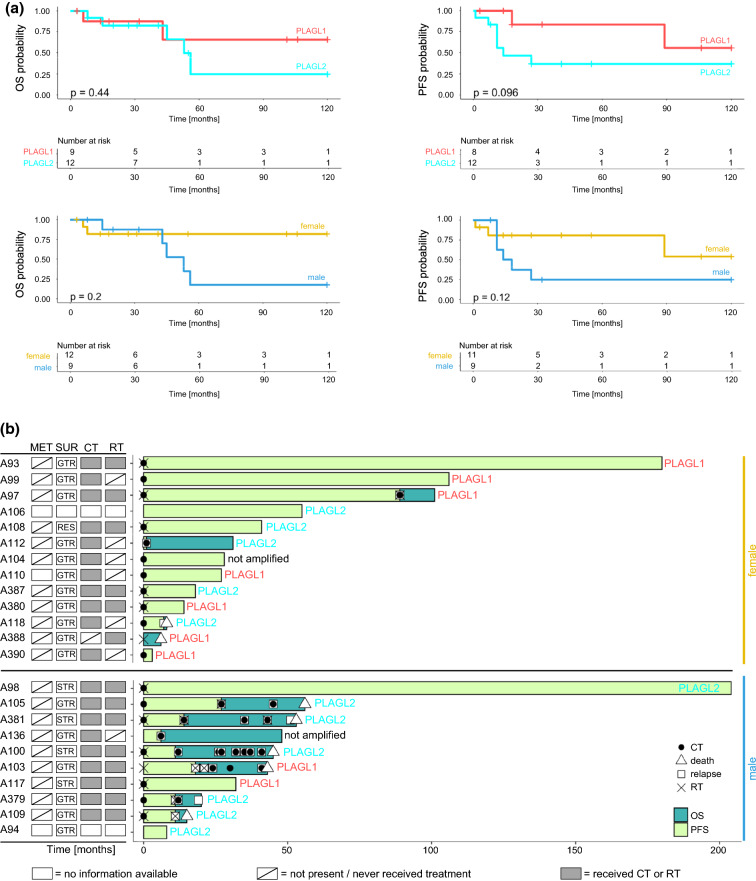
Clinical outcomes of patients with CNS embryonal tumor with PLAGL gene amplification. **a** Kaplan–Meier plots showing OS and PFS stratified by subgroup and sex. The log-rank test was used to show differences between the curves, p-values of the log-rank test are shown in each graph. **b** Swimmer plot showing available OS and PFS times per patient, including treatment information and clinical response/relapse. Samples are stratified by sex, *PLAGL1/2* amplification status is indicated. Information about surgical resection (SUR) and presence of metastasis (MET) at the time point of primary diagnosis is displayed in the squares on the left where available (resections or metastases at later time points are not displayed), GTR, gross total resection; STR, subtotal resection; RES, resection (unknown, if GTR or STR). Information about chemotherapy (CT) and radiotherapy (RT) treatment regarding the entire follow-up time is displayed in the squares on the left where available

The original article has been corrected.

