# Supplementary material for: Amplification of the PLAG-family genes—PLAGL1 and PLAGL2—is a key feature of the novel tumor type CNS embryonal tumor with PLAGL amplification
Source: Acta Neuropathol. 2022 Nov 27;145(1):49–69. doi: 10.1007/s00401-022-02516-2 (PMC9807491; doi:10.1007/s00401-022-02516-2)
Supplement: Supplementary file 2 — Supplementary file2 (PDF 35368 KB) [file 401_2022_2516_MOESM2_ESM.pdf]

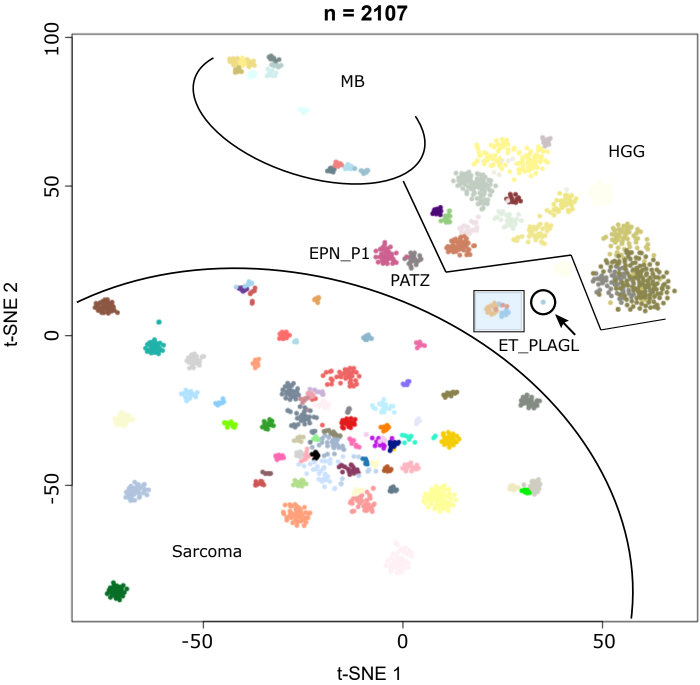

Supplementary Figure 1. DNA methylation-based analysis using t-SNE dimensionality reduction on 33 embryonal tumors with *PLAGL*-amplification (ET, PLAGL) and the reference cohort of 910 CNS tumors that was used for figure 1b. In this t-SNE plot 1,124 sarcomas (Sarcoma) and 40 supratentorial ependymoma-like tumors with *PLAGL1* fusions (EPN_P1) were added in addition. Methylation classes are color-coded. PATZ = neuroepithelial tumor with *PATZ1* fusion. ET, PLAGL tumors are surrounded with a square and differentially colored according to their amplified PLAG-family gene. Two outlying *PLAGL*-amplified samples are circled and marked with an arrow.

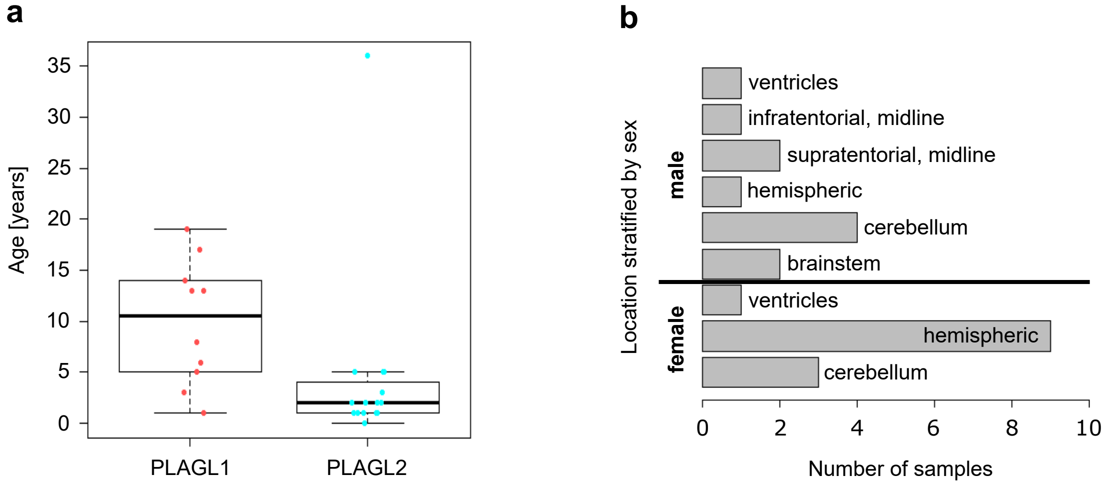

Supplementary Figure 2. Patient characteristics were visualized for the cohort of 31 ET, PLAGL samples. **a** Boxplots showing the age range and median age for the *PLAGL1*- and *PLAGL2*-amplified samples respectively **b** Barplots showing the anatomic location of ET, PLAGL tumors stratified by sex.

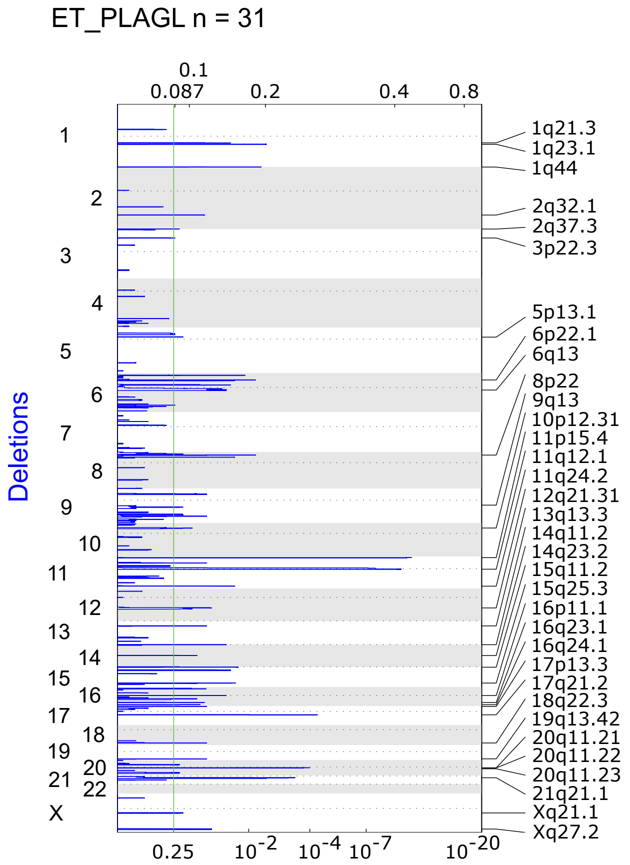

Supplementary Figure 3. GISTIC deletion plots of all 31 samples belonging to the ET, PLAGL type. The genome is displayed vertically on the y-axis and genomic positions of amplified regions are indicated. Normalized amplification signals (G-score) and q-values (log scale) are indicated on the X-axis on the top and bottom, respectively. The green line represents the significance cutoff (q-value=0.25).

Patient A388, 13 y/o F, *PLAGL1* amplified

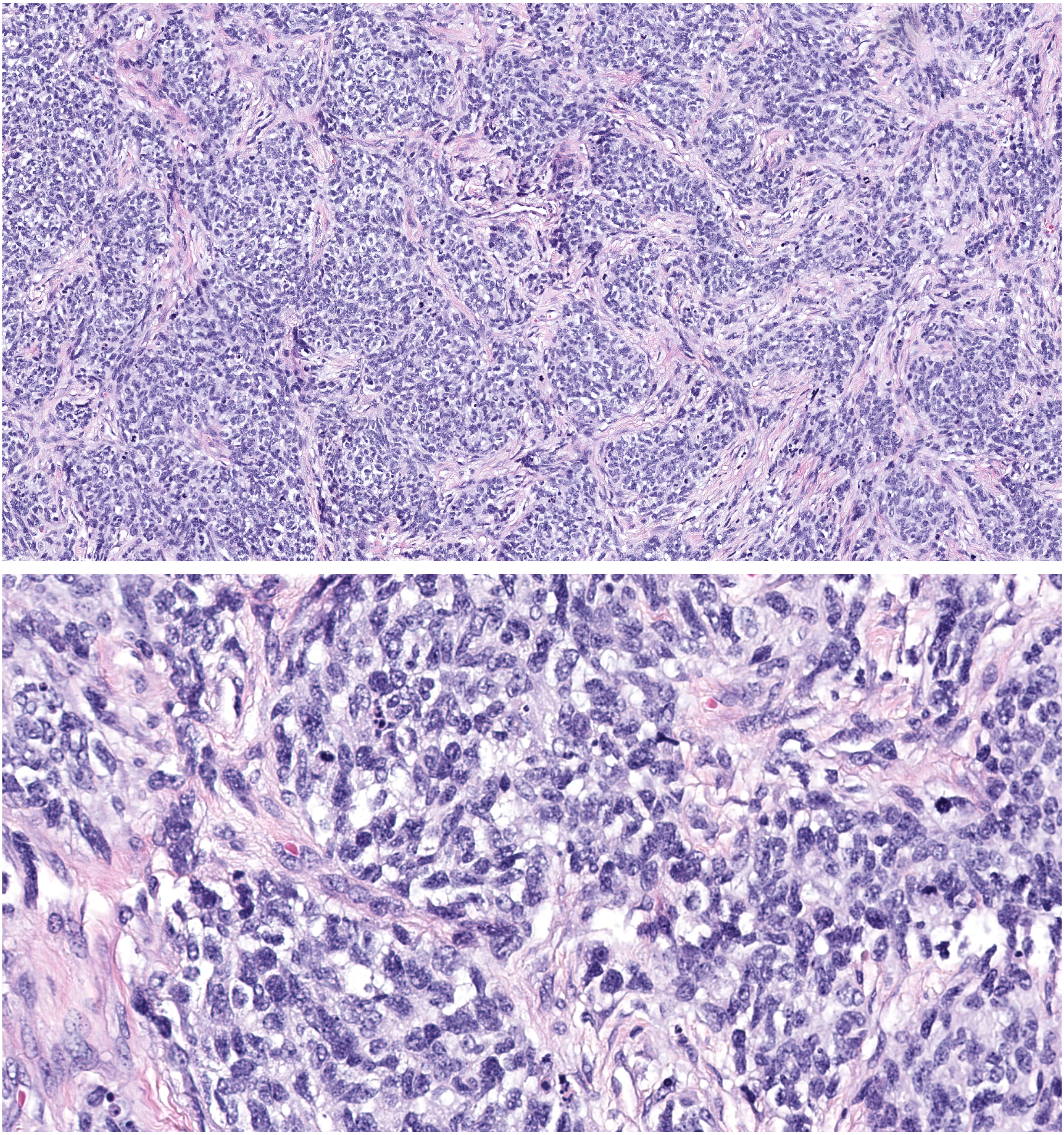

Supplementary Figure 4. Histology of CNS embryonal tumors with PLAGL gene amplification. Shown are representative H&E stained sections of tumors with *PLAGL1* or *PLAGL2* amplification at low magnification (top panels) and high magnification (bottom panels).

Patient A112, 1 y/o F, *PLAGL2* amplified

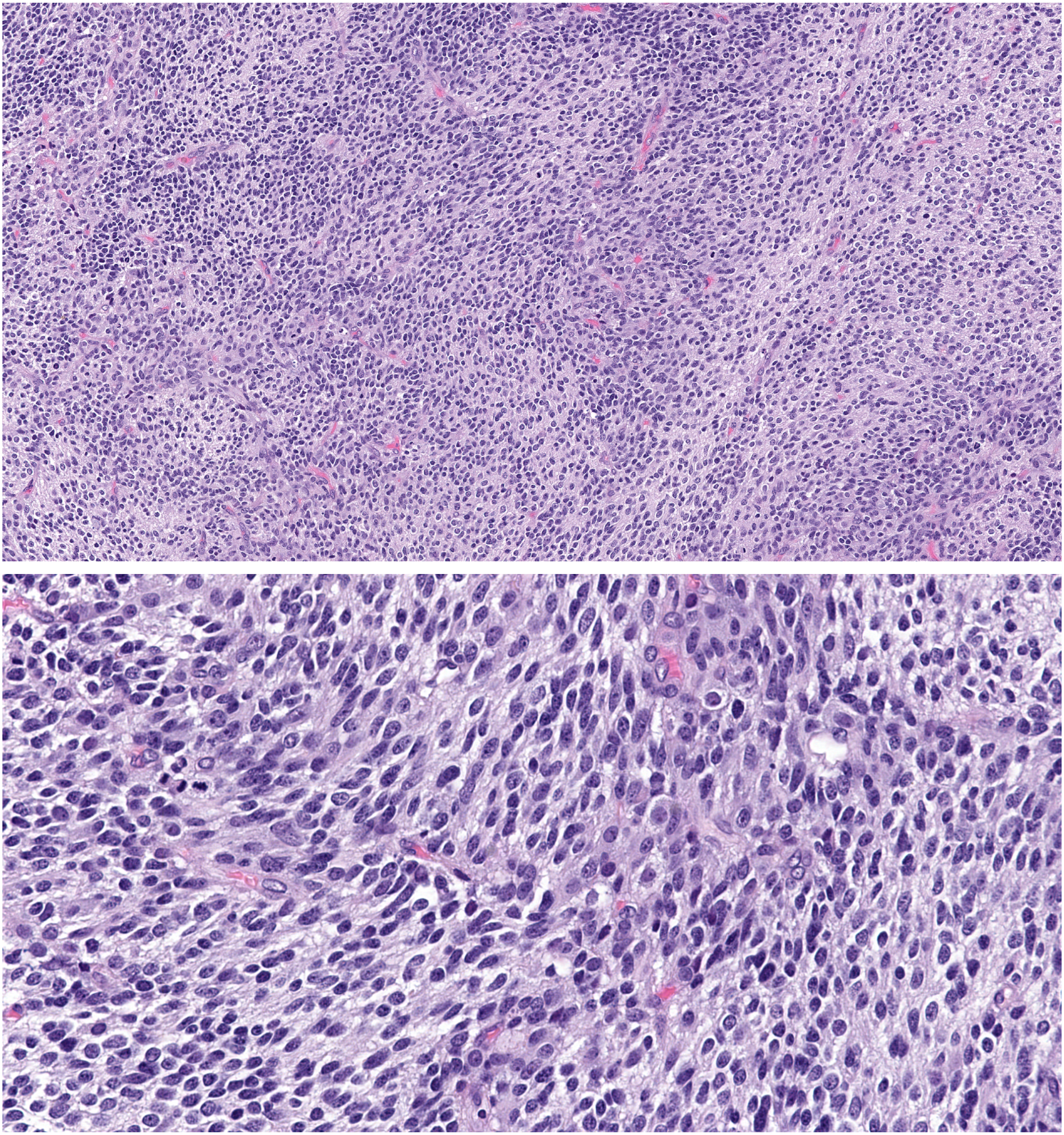

Supplementary Figure 4 continued

Patient A113, 2 y/o F, *PLAGL2* amplified

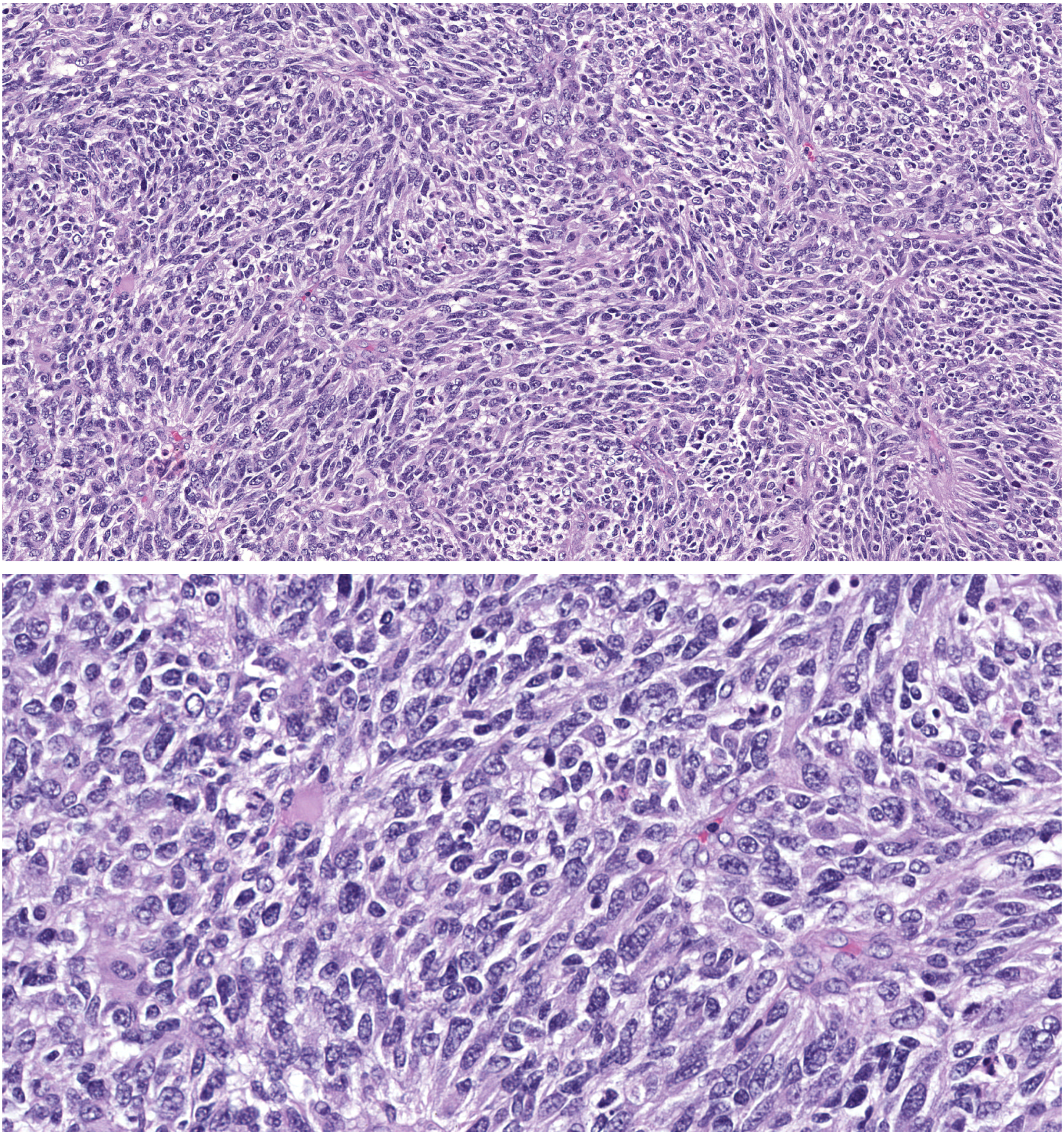

Supplementary Figure 4 continued

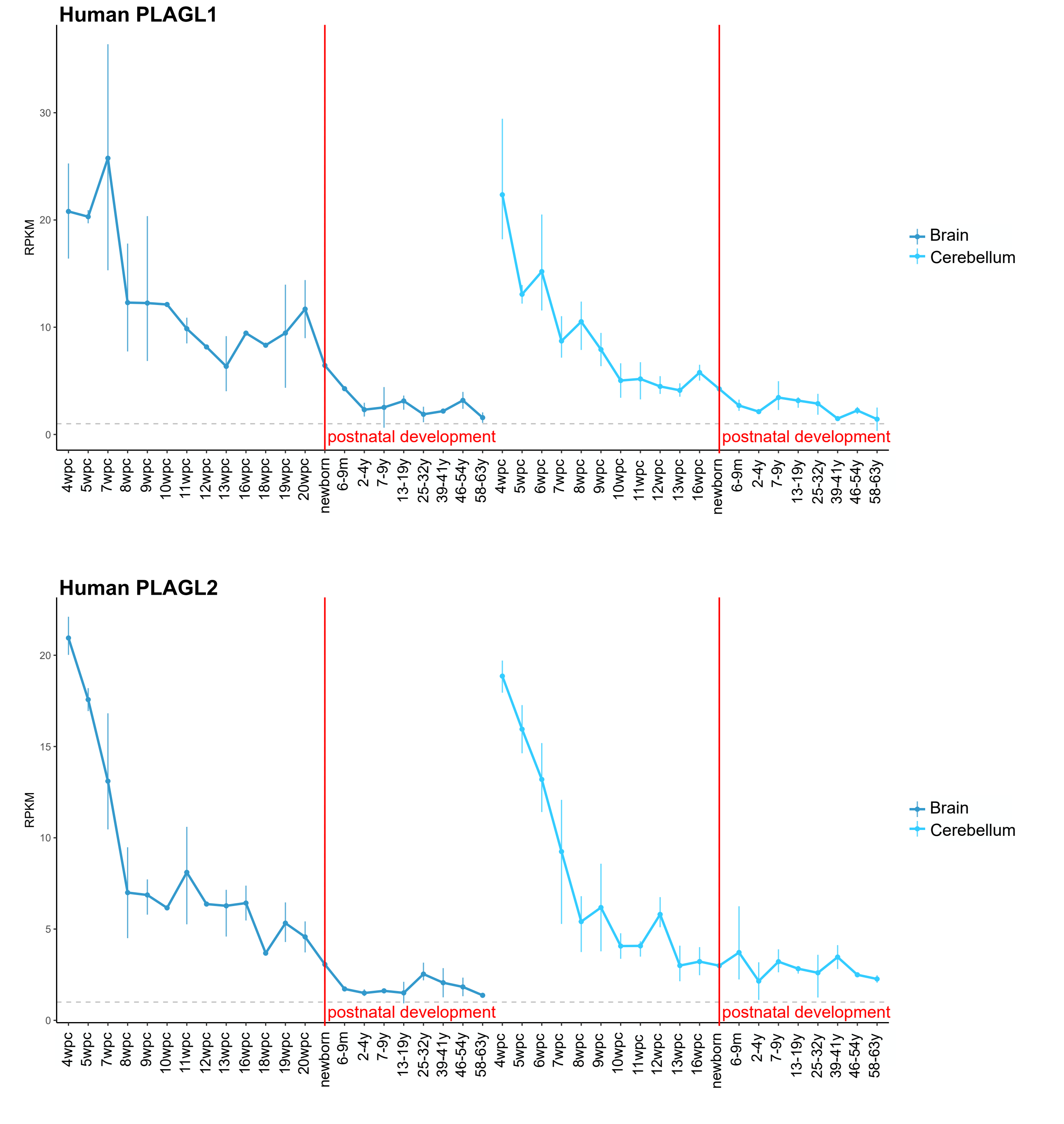

Supplementary Figure 5. Expression levels of human PLAGL1 and PLAGL2 in brain (forebrain) and cerebellum (hindbrain) during prenatal and postnatal development as created with the evo-devo app by the Kaessmann lab (https://apps.kaessmannlab.org/evodevoapp/) [13].

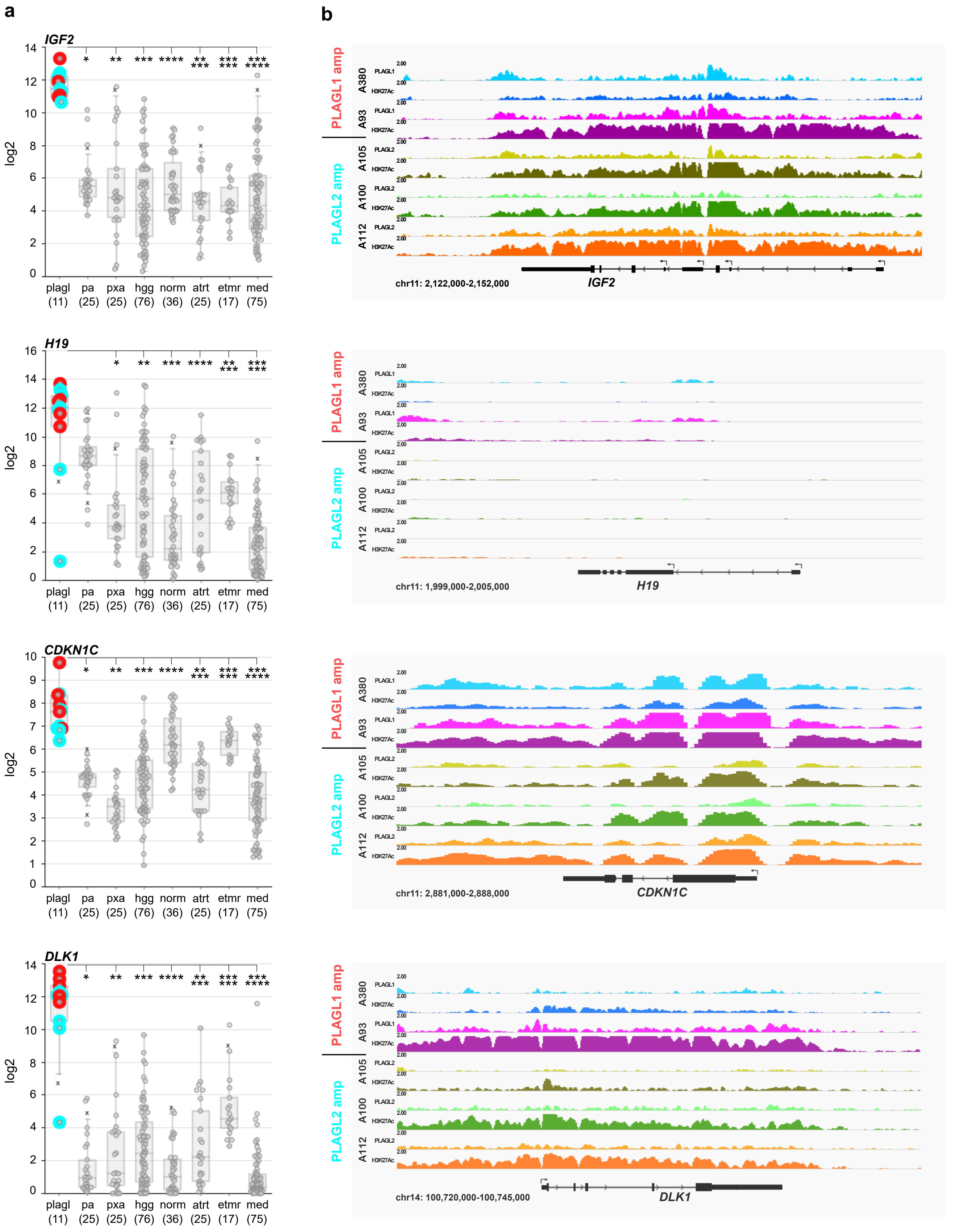

Supplementary Figure 6. a Boxplots comparing gene expression between CNS tumor types (n = 290 samples) for a subset of IGs. Red: Samples with *PLAGL1*-amplification, blue: Samples with *PLAGL2*-amplification. Significance bars indicate groups whose differences in gene expression are statistically significant when compared to samples with *PLAGL1/2*-amplification (t-test, Bonferroni-corrected p-value = 0.00714286). plagl = ET,PLAGL; pa = pilocytic astrocytoma; pxa = pleomorphic xanthoastrocytoma; hgg = high grade gliomas (G34R/V, K27M, pedRTK1/2); norm = normal brain tissues; atrt = atypical teratoid rhabdoid tumor; etmr = embryonal tumor with multilayered rosettes; med = medulloblastomas (WNT, SHH, group 3, group 4); **b** ChIPseq traces for PLAGL1, PLAGL2 and H3K27ac ChIPs. PLAGL1 ChIPs were performed in *PLAGL1*-amplified samples, PLAGL2 ChIPs were performed in *PLAGL2*-amplified samples, H3K27ac ChIPs show that PLAGL binding is related to a specific enhancer region and is potentially functional as a regulator. Further indicated are the respective genes, chromosomal region and signal intensity.

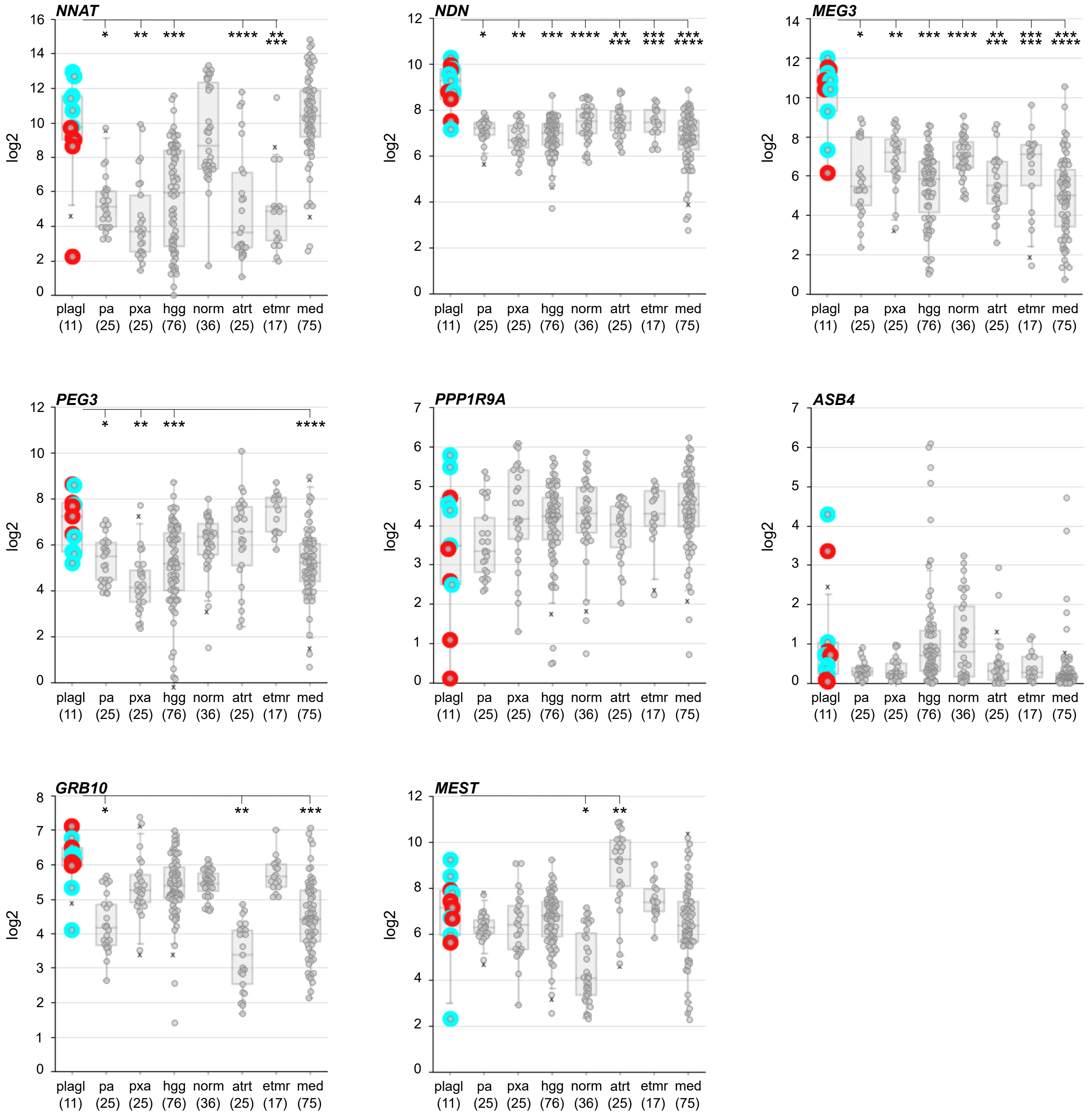

Supplementary Figure 7. Boxplots comparing gene expression between CNS tumor types (n = 290 samples) for a further subset of IGs. Red: Samples with *PLAGL1*-amplification, blue: Samples with *PLAGL2*-amplification (t-test, Bonferroni-corrected p-value = 0.00714286). Significance bars indicate groups whose differences in gene expression are statistically significant when compared to samples with *PLAGL1/2*-amplification. plagl = ET,PLAGL; pa = pilocytic astrocytoma; pxa = pleomorphic xanthoastrocytoma; hgg = high grade gliomas (G34R/V, K27M, pedRTK1/2); norm = normal brain tissues; atrt = atypical teratoid rhabdoid tumor; etmr = embryonal tumor with multilayered rosettes; med = medulloblastomas (WNT, SHH, group 3, group 4);

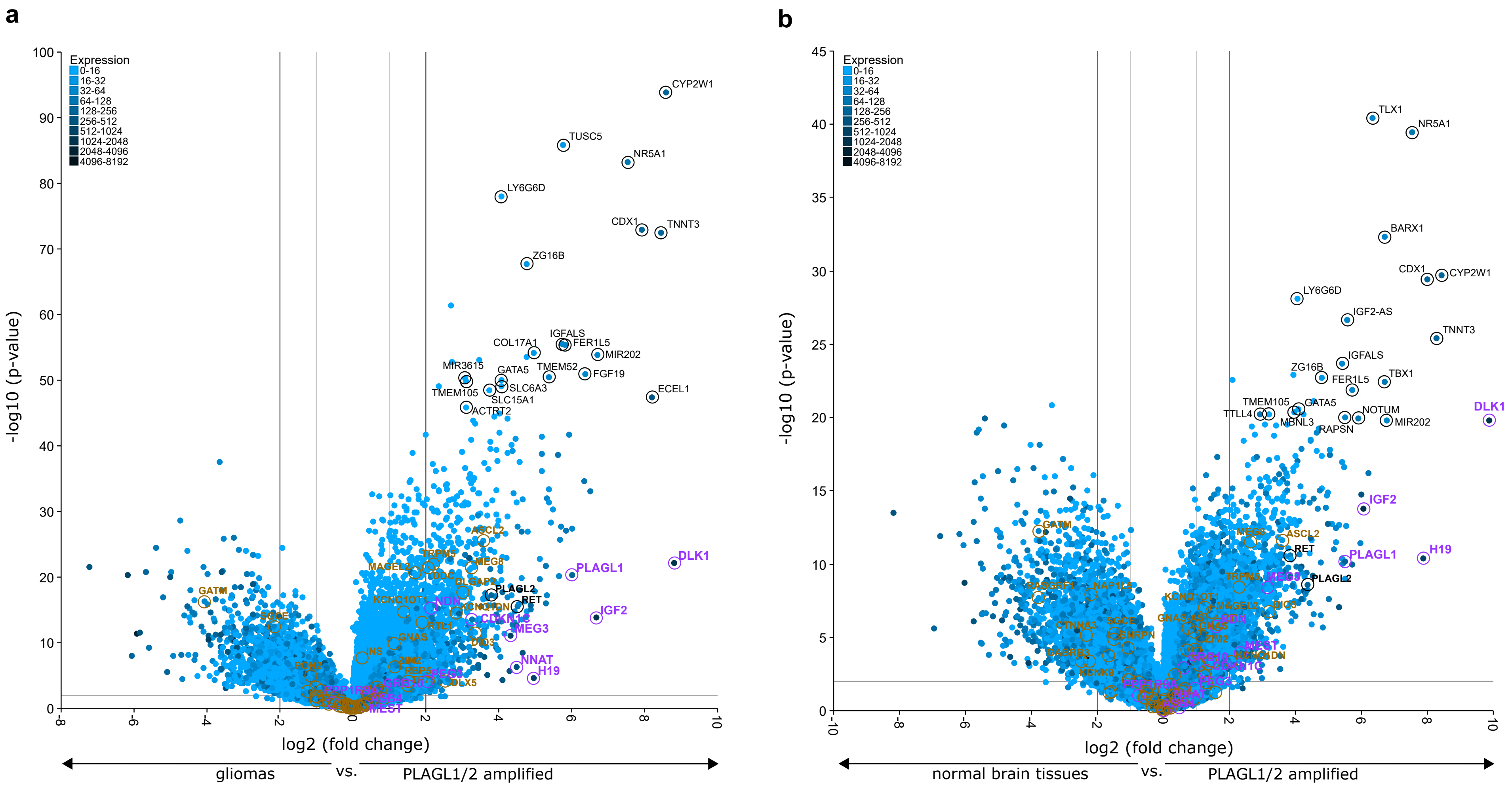

Supplementary Figure 8. Gene expression profiles of CNS embryonal tumors with PLAGL gene amplification. **a, b** Volcano plots showing fold-change and p-value for the comparison of differential gene expression of 11 PLAGL1/2-amplified tumors versus **a** different glial tumor types (n = 126) and **b** various adult and fetal normal brain tissues (n = 36). Highlighted are 86 human IGs (ocher) and 13 IGs with high connectivity (lilac) as described in reference [5]. Shown in black: selection of genes with large magnitude fold-changes (x axis) and high statistical significance (-log10 of p value, y axis).

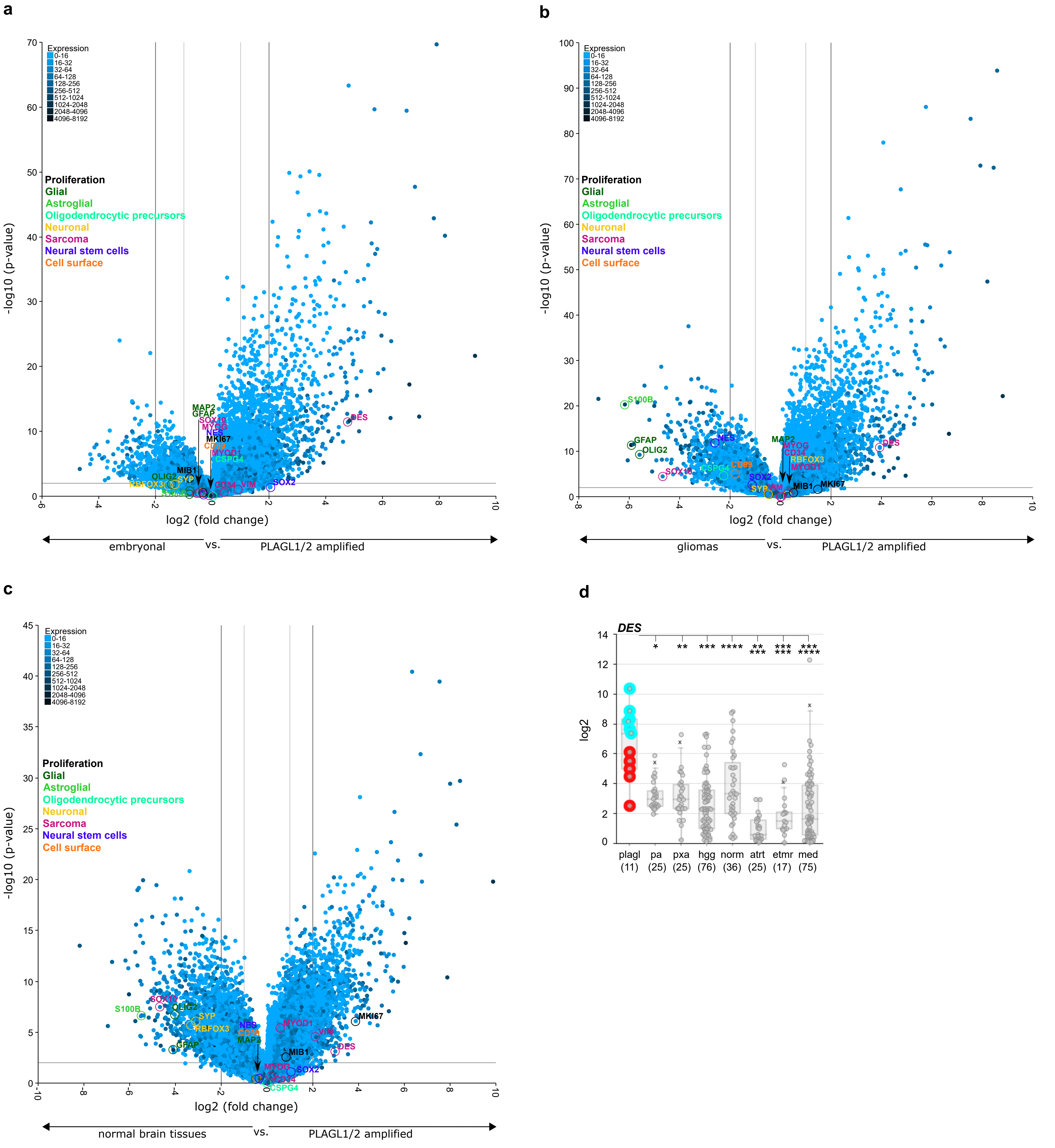

Supplementary Figure 9. a, b, c Volcano plots showing fold-change and p-value for the comparison of differential gene expression of 11 *PLAGL1*/*PLAGL2*-amplified tumors versus **a** 117 embryonal tumors **b** 126 gliomas and **c** 36 fetal and adult normal brain tissues. Highlighted are classical pan neuronal, glial, sarcoma/mesenchymal, neural stem cell, and proliferation marker genes. **d** Boxplot comparing *Desmin* expression between CNS tumor types. Samples (n=290) are identical to a+b+c. Red: Samples with *PLAGL1*-amplification, blue: Samples with *PLAGL2*-amplification. Significance bars indicate groups whose differences in gene expression are statistically significant when compared to samples with *PLAGL1/2*-amplification (t-test, Bonferroni-corrected p-value = 0.00714286). plagl = ET,PLAGL; pa = pilocytic astrocytoma; pxa = pleomorphic xanthoastrocytoma; hgg = high grade gliomas (G34R/V, K27M, pedRTK1/2); norm = normal brain tissues; atrt = atypical teratoid rhabdoid tumor; etmr = embryonal tumor with multilayered rosettes; med = medulloblastomas (WNT, SHH, group 3, group 4);

a

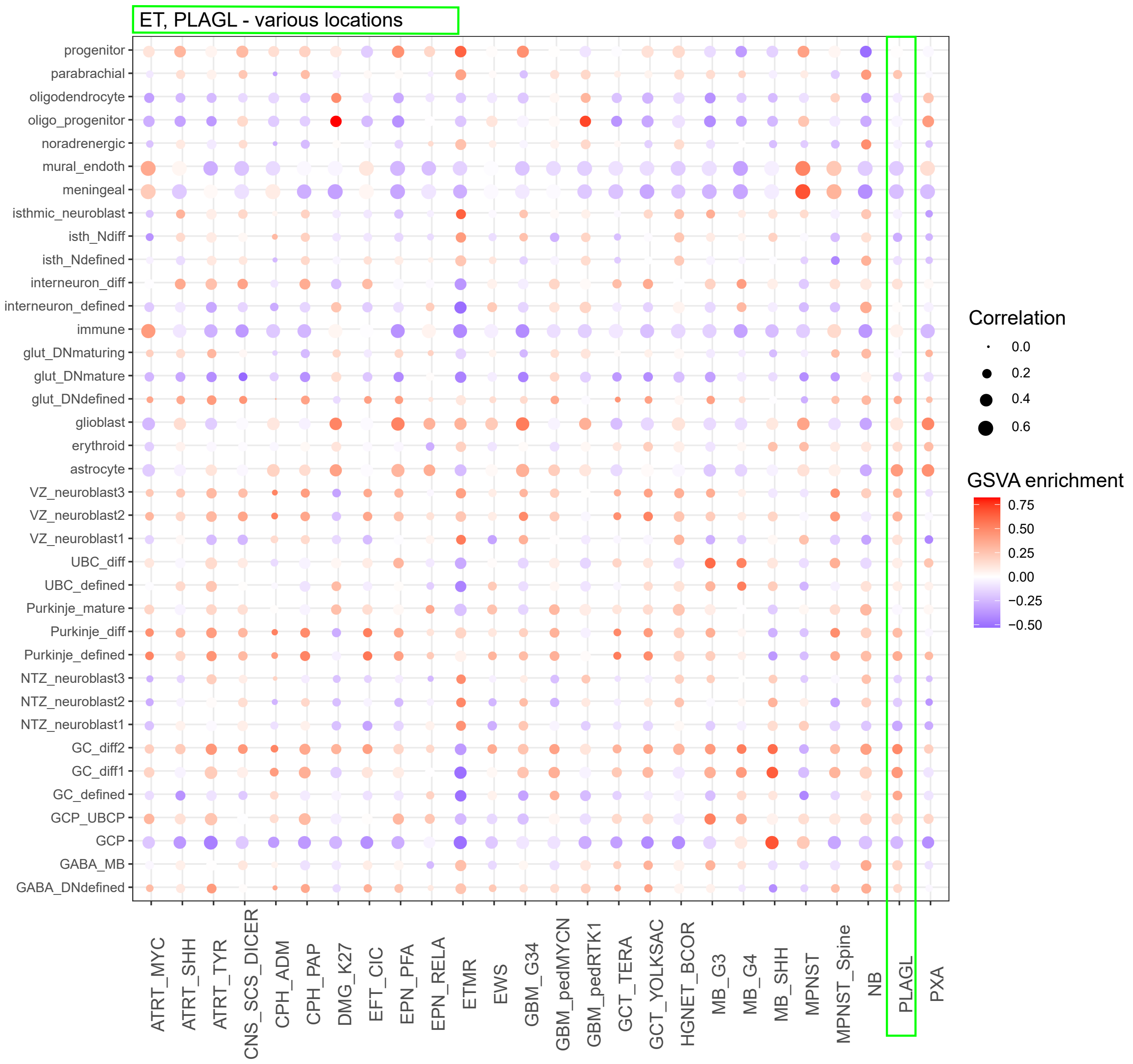

b

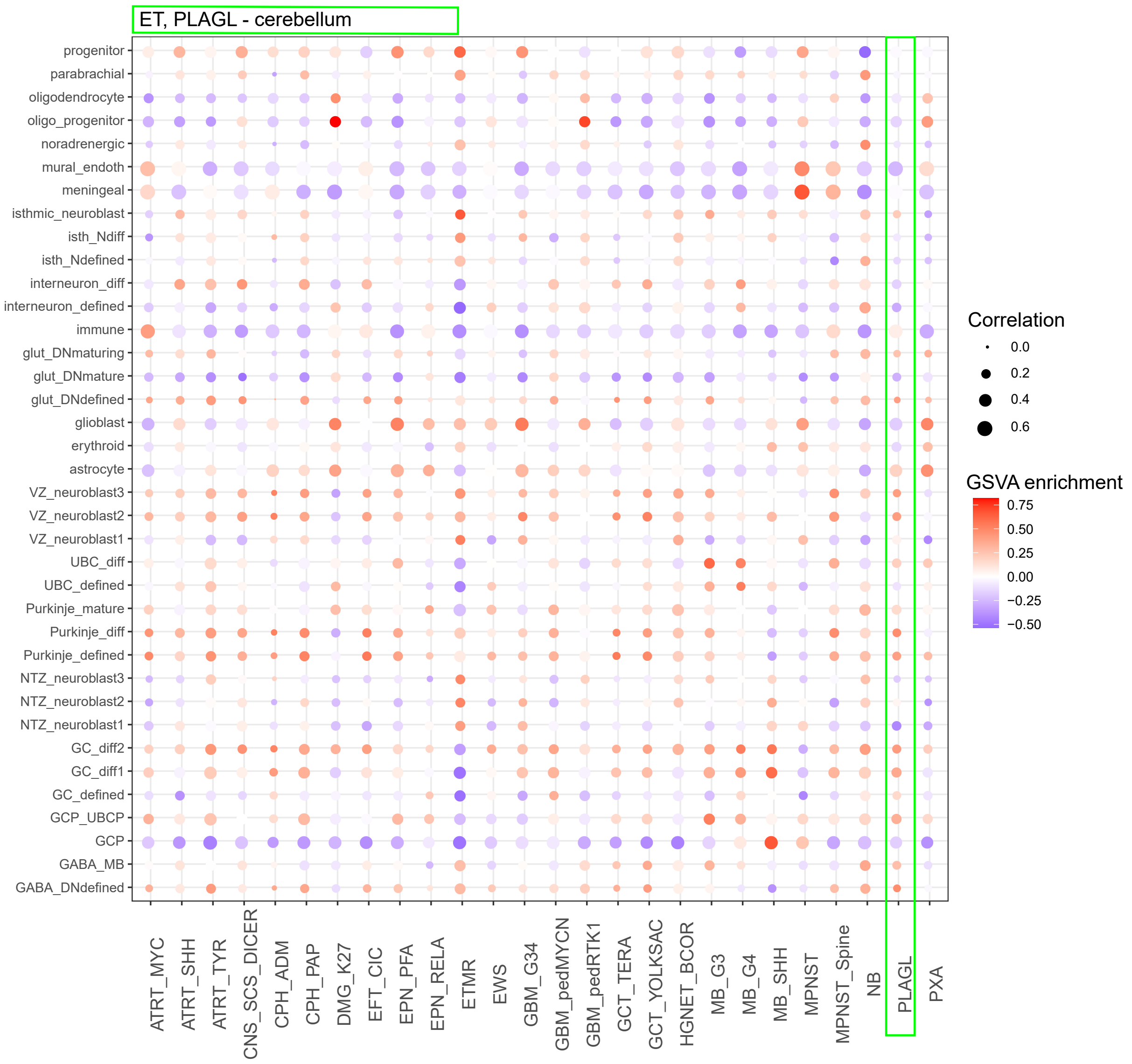

Supplementary Figure 10. Heatmap showing comparisons of CNS tumor bulk gene expression profiles (in columns) to cerebellar development cell state (in rows), based on gene signature enrichment score via Gene Set Variance Analysis (GSVA enrichment) indicated by color and Pearson correlation score (Correlation) indicated by area. **a** Comparison was done using bulk expression data of ET, PLAGL tumors (PLAGL, marked with the green frame) from various anatomic locations (n = 11). **b** Comparison was done using bulk expression data of cerebellar ET, PLAGL tumors (PLAGL, marked with the green frame, n = 4).

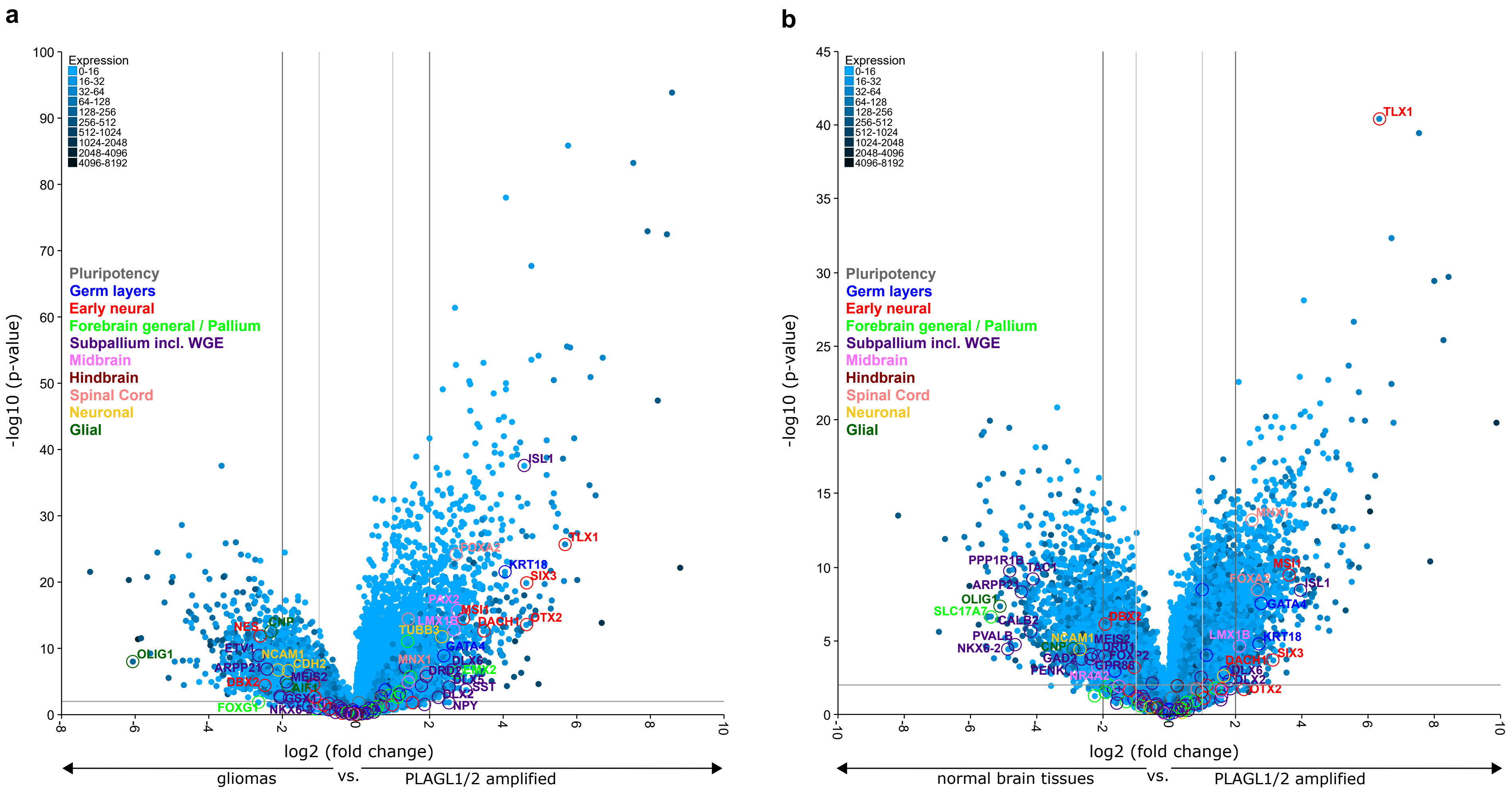

Supplementary Figure 11. Gene expression profiles of CNS embryonal tumors with PLAGL gene amplification. Volcano plots showing fold-change and p-value for the comparison of differential gene expression of 11 PLAGL1/2-amplified tumors versus **a** different glial tumor types (n = 126) and **b** various adult and fetal normal brain tissues (n = 36). Highlighted are genes with differential expression in different brain regions and during different developmental states as described in reference [56].

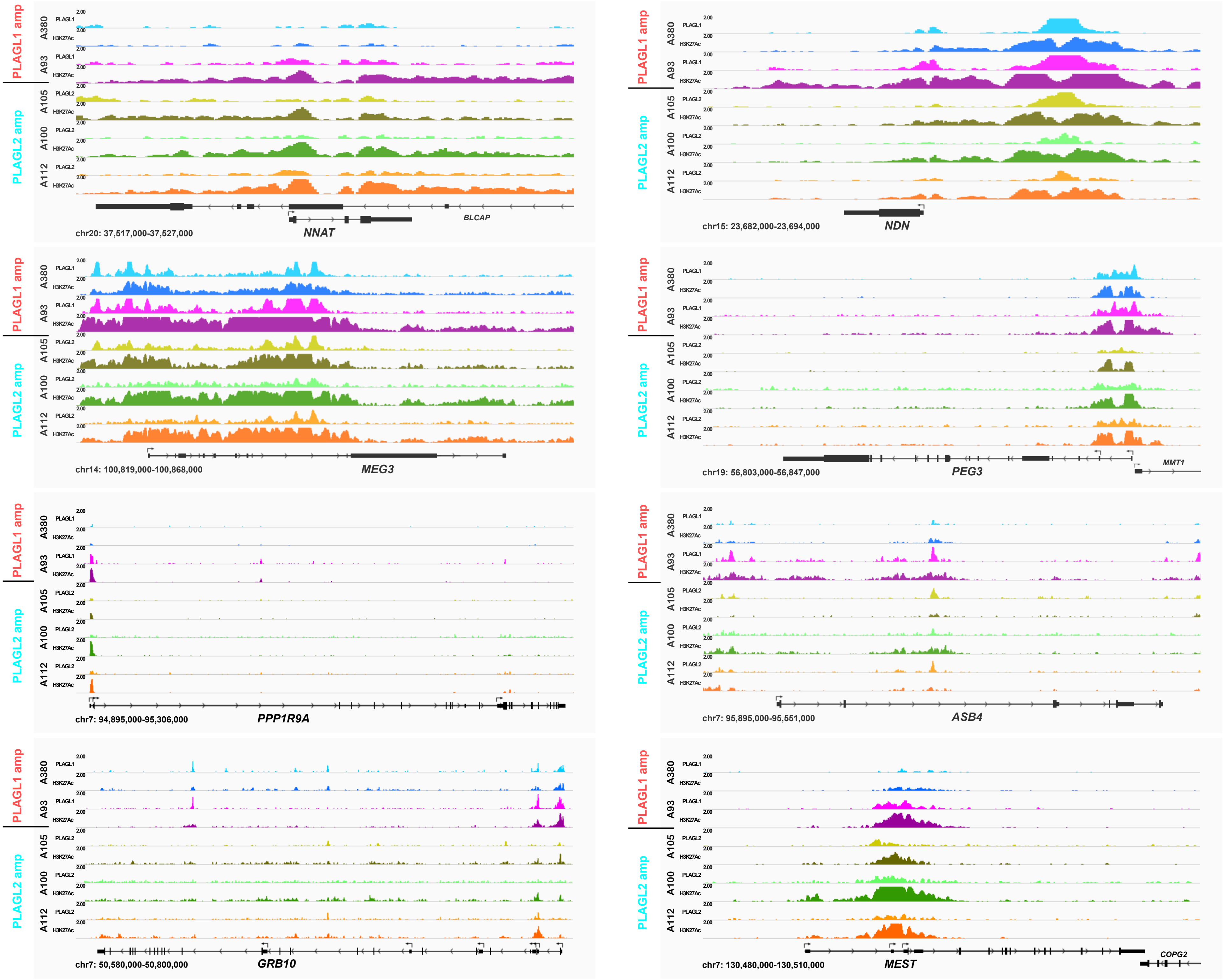

Supplementary Figure 12. ChIPseq traces for PLAGL1, PLAGL2 and H3K27ac ChIPs. PLAGL1 ChIPs were performed in *PLAGL1*-amplified samples, PLAGL2 ChIPs were performed in *PLAGL2*-amplified samples, H3K27ac ChIPs show that PLAGL binding is related to a specific enhancer region and is potentially functional as a regulator. Further indicated are the respective genes, chromosomal region and signal intensity.

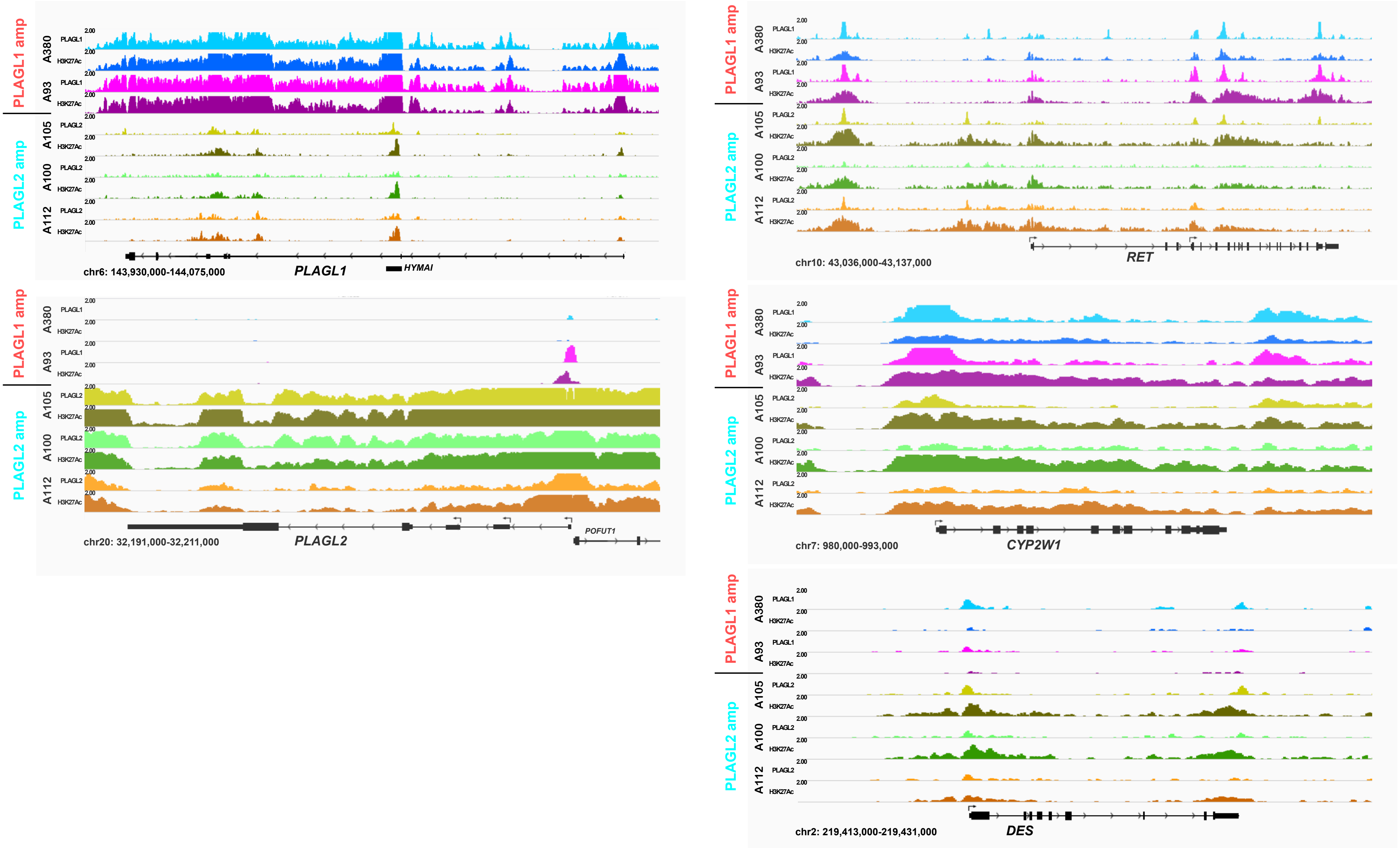

Supplementary Figure 13. ChIPseq traces for PLAGL1, PLAGL2 and H3K27ac ChIPs. PLAGL1 ChIPs were performed in *PLAGL1*-amplified samples, PLAGL2 ChIPs were performed in *PLAGL2*-amplified samples, H3K27ac ChIPs show that PLAGL binding is related to a specific enhancer region and is potentially functional as a regulator. Further indicated are the respective genes, chromosomal region and signal intensity.

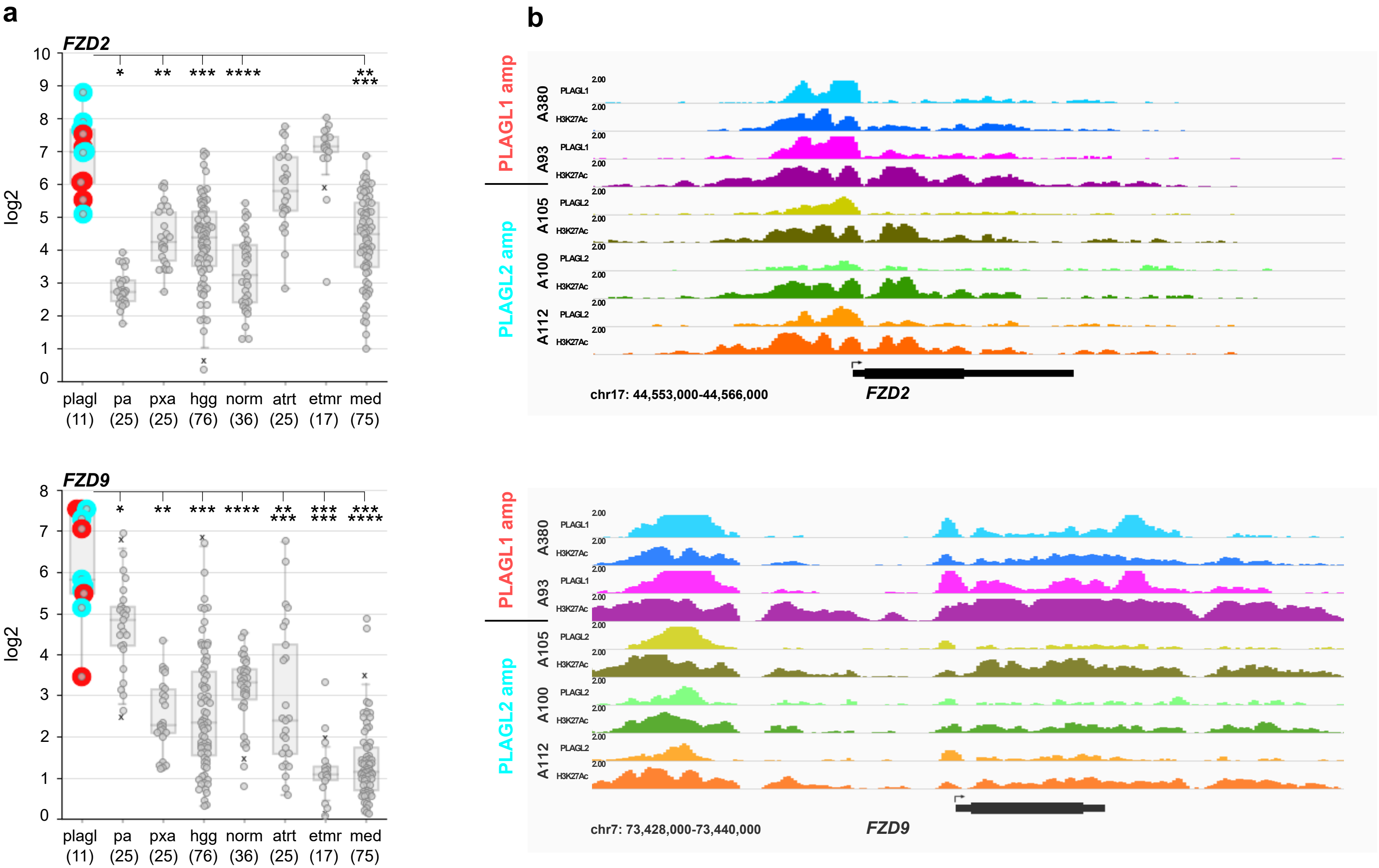

Supplementary Figure 14. a Boxplots comparing gene expression between CNS tumor types (n = 290 samples) for a subset of genes involved in β-Catenin/Wnt-signaling. Red: Samples with *PLAGL1*-amplification, blue: Samples with *PLAGL2*-amplification. Significance bars indicate groups whose differences in gene expression are statistically significant when compared to samples with *PLAGL1/2*-amplification (t-test, Bonferroni-corrected p-value = 0.00714286). plagl = ET,PLAGL; pa = pilocytic astrocytoma; pxa = pleomorphic xanthoastrocytoma; hgg = high grade gliomas (G34R/V, K27M, pedRTK1/2); norm = normal brain tissues; atrt = atypical teratoid rhabdoid tumor; etmr = embryonal tumor with multilayered rosettes; med = medulloblastomas (WNT, SHH, group 3, group 4); **b** ChIPseq traces for PLAGL1, PLAGL2 and H3K27ac ChIPs. PLAGL1 ChIPs were performed in *PLAGL1*-amplified samples, PLAGL2 ChIPs were performed in *PLAGL2*-amplified samples, H3K27ac ChIPs show that PLAGL binding is related to a specific enhancer region and is potentially functional as a regulator. Further indicated are the respective genes, chromosomal region, and signal intensity.

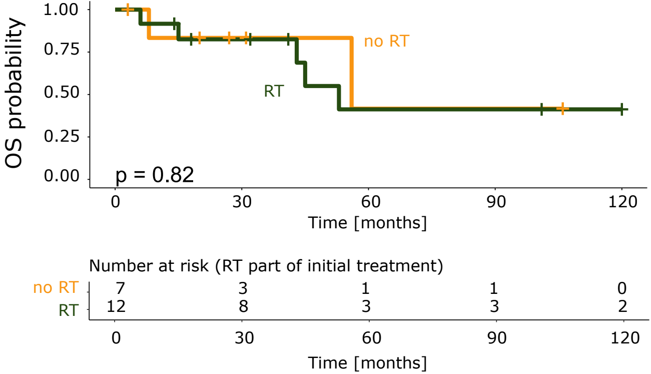

Supplementary Figure 15. Kaplan-Meier plots showing OS stratified by treatment, RT = radiotherapy was part of initial treatment, no RT = radiotherapy was not included in the initial treatment of the patient. The log-rank test was used to show differences between the curves, p-value of the log-rank test is shown in the graph.
